# Lactation in quarantine: The (in)visibility of human milk feeding during the COVID-19 pandemic in the United States

**DOI:** 10.1186/s13006-022-00451-2

**Published:** 2022-03-21

**Authors:** Mathilde Cohen, Corinne Botz

**Affiliations:** 1 University of Connecticut School of Law, Connecticut, USA; 2 Visual Artist, New York, USA

**Keywords:** Breastfeeding, Lactation, Pandemic, COVID-19, Gender inequality, Parenting, Human milk feeding, Milk sharing, Donor human milk

## Abstract

**Background:**

In response to the COVID-19 pandemic, billions of people were asked by their state and local governments not to go to work and not leave the house unless they had to. The goal of this qualitative study was to collect the lived experiences of a small group of parents and lactation professionals in the United States about what it was like to feed babies human milk under these conditions of quarantine.

**Methods:**

This project is a social constructionist analysis of lactation narratives of 24 parents feeding their children human milk and 13 lactation professionals. They were interviewed remotely in 2020–21 via videoconferencing about their experiences and perspectives on the pandemic’s effect on lactation. Additionally, photographs of 16 of the parents are provided to visualize their practices and how they chose to represent them.

**Results:**

Four interrelated themes were identified in participants’ narratives about how they experienced and made sense of human milk feeding during the pandemic: the loneliness of lactation during the pandemic, the construction of human milk as a resource to cope with the crisis, the (in)visibility of lactation amidst heightened multitasking, and the sense of connection created by human milk feeding at a time of unprecedented solitude.

**Conclusions:**

While the pandemic may have had both positive and negative effects on lactation, it exposed continuing inequities in infant feeding, generating new forms of (in)visibility for lactating labor. Going forward, one lesson for policy and lawmakers may be that to adequately support lactation, they should take cues from the families who had positive experiences during the crisis. This would call for systemically overhauling of US laws and policies by guaranteeing: universal basic income, paid parental leave for at least six months, paid lactation leaves and breaks, affordable housing, universal health care, subsidized childcare programs, and equal access to high-quality, non-discriminatory, and culturally appropriate medical care—including lactation counseling—, among other initiatives.

## Background

Two years into the COVID-19 pandemic, awareness is growing that the crisis proved challenging in various ways for human milk feeding. (Note that we understand “human milk feeding” broadly to mean feeding at the nipple as well as feeding a child expressed human milk [1].) Some early COVID-19 policies led to the separation of infants from their parents at the hospital, preventing or impeding lactation despite a lack of evidence of vertical transmission of SARS-CoV-2 [2–4]. In the United States, these practices seem to have disproportionately affected and harmed Black and Indigenous families [5, 6]. Lactation services were disrupted, with decreased of in-person lactation assistance and shift to virtual or modified formats [7, 8]. Some parents changed their infant feeding plans due to the crisis, be it by stopping to breastfeed earlier than they had planned [2] or by deciding to breastfeed instead of using infant formula because of formula shortages or the belief that human milk would better protect their babies from infection [9, 10]. Postpartum parents generally experienced more challenges with mental health and stress due to the isolation associated with the management of the crisis [11, 12], which in some case prompted them to formula feed [10]. Multiple accounts have highlighted the increased responsibilities weighing on American parents, particularly mothers, due to the prolonged closure of daycares and schools and the breakdown of many other sources of support [13, 14], which may have created further obstacles to lactation.

Some experts worry that due to COVID-19, “both breastfeeding initiation and duration rates are at much risk for declining globally” [15]. While 84.1 percent of American babies are breastfed at least once, only about one in four are breastfed exclusively for six months in contradiction to medical and public health recommendations [16]. Due to the multiple barriers in the path of Black and Native American women in particular fewer Native and Black infants are ever breastfed compared with Asian infants, Latinx infants, and White infants [17, 18]. There is concern that disparities in human milk feeding along factors such as race/ethnicity, age, educational attainment, and income worsened with the pandemic [5–7, 19, 20].

Despite the growing availability of scientific and public information on lactation and COVID-19, the experiences and narratives of parents feeding their children human milk and of lactation professionals trying to support them remain in the background. Yet, qualitative research about the impact of the pandemic on parents’ lactation practices, expectations, and perceptions of social support is crucial not only to develop improved responses to future crises, but also to inform postpartum guidelines and policies in ordinary times.

## Methods

This article combines the person-centered and narrative traditions of qualitative research with some elements of visual ethnography [21, 22]. Research began in April of 2020, animated with the question: how do parents and lactation professionals practice and think about human milk feeding, which epitomizes social closeness and embodied interrelatedness, during a crisis that precipitated quarantines, social distancing, and suspicion around bodily fluids? Because lactation felt even more invisible than usual during a time when non-essential workers were asked to stay home, we teamed up to attend to its narration and visual representation in the conditions of quarantine. As two White cishet middle-class women who work on human milk feeding, we wanted to explore and convey the stories and images of parents raising and feeding young children in our current moment. While we were both quarantined with our respective families, we engaged in open-ended, multi-sited, and collaborative research/art. Cohen conducted in-depth interviews with parents and lactation specialists through videoconferencing while Botz took remote pictures of those of the parents desiring to be photographed. This project is not auto-ethnography as it does not center on the self, but it is a reflexive study relying on active interviewing [23] as its primary source of data and photographs taken collaboratively by Botz and some of the participants to provide visual context.

The interviews were carried out between the spring of 2020 and the spring of 2021 via videoconferencing with 37 participants: 24 were parents feeding their children human milk and 13 were lactation specialists (3 of the 24 parents were also lactation professionals). Some of the parents were feeding at the breast, some were exclusively pumping, some were using donor human milk, and some combined one or more of these feeding methods. Lactation specialists included a midwife, several lactation consultants (IBLCLs and CLCs), a neonatologist, and a breastfeeding advocate. All participants were US residents, except three who lived in Canada and one in Europe at the time of the interview. All participants identified as women, but for one parent who identified as a man/father. Four parents out of the 24 were unemployed, be it by choice or by force. Two were single, while the others were all partnered. All were middle or upper-middle class except for two low-income participants. Thirteen parents identified as White, 5 as Black, 4 as Latinx, and 1 as Arab/Muslim. Ten lactation specialists identified as White, one as Black, one as Latina, and one as Native-American. Throughout the essay, we specify the race/ethnicity of research participants the first time we mention them, using the identity categories they chose.

The question whether and how to anonymize participants proved vexing given that but for two informants who preferred to remain anonymous, participants wanted their names, stories, and appearance (when their photographs were taken) to be public. They hoped to bring greater recognition to the challenges of lactation during the pandemic, which they deemed insufficiently acknowledged. As Bonnie Scarth has argued “anonymity becomes problematic when participants want to be identified for various reasons” [24]. Despite our goal of furthering participants’ desire for agency and identity, following feedback from this journal’s peer-reviewers asking us to anonymize, we concluded that participants’ hope of making lactation in quarantine more knowable and visible would be realized even if their real names were not used. We thus use pseudonyms with the idea that participants who wanted most exposure would still be recognizable in the photographs.

In the traditional tradition of qualitative sampling, a combination of snowball sampling and purposive sampling was used to contact participants and maximize variations in backgrounds as well as in lactation experience. Through our social networks, we obtained referrals for potential participants. A research project description was posted on Facebook as well as on an online parenting and breastfeeding group. The inclusion criteria for parents were: wanting to share their stories of feeding their children human milk during the pandemic (or of being unable do so). For lactation specialists, the criteria were: professional involvement in lactation, be it as a specifically trained lactation consultant, a peer counselor, or a health professional serving pregnant and postpartum parents and/or infants. Data saturation was achieved by the fifteenth interview for parents and the tenth for lactation specialists. The interviews were unstructured, narrative interviews, conducted by Cohen, lasting between 30–90 min. Ethical approval was obtained from the University of Connecticut IRB and informed written consent was obtained for each interview and photo shoot participant. Most interviews were recorded and transcribed. Transcripts were not returned to participants for comments.

We used feminist [25] and active interviewing [23], that is, qualitative approaches that recognize that researchers and participants both play active roles in the narrative production and strive to share or negotiate interpretive authority. Interview questions focused on parents’ experience initiating lactation (if applicable), work and childcare obligations, impact of the pandemic, and demographic information. For lactation specialists, the interview started with the question “How has COVID-19 affected your work?” Follow-up questions included, “How do you think COVID-19 affected lactation?” and “What laws and policies would you like to see in place to support lactation?” These were general questions to guide the conversations, letting participants control the direction, content, and pace of the interview.

The analysis consisted of general and focused coding [21] to identify how participants experienced and made meaning of lactation during the pandemic. We subjected the interview transcripts and notes to content analysis as soon as the data collection began. We identified themes and categories with the first few interviews, which were used to inform following interviews. We did not use any software, but wrote analytic memos as we coded, discerning conceptual themes across cases. We then conducted more focused coding on the full dataset, examining the similarities and differences within each theme. We distinguished four interrelated themes as the most prominent around the feeling of isolation, overwhelm, and lack of recognition—the loneliness of pandemic breastfeeding, the construction of human milk as a resource to cope with the crisis, the (in)visibility of lactation amidst heightened multitasking, and the sense of connection created by human milk feeding at a time of unprecedented solitude. To increase content validity, we read the transcripts and our notes multiple times to analyze the text.

**Fig. 1:**
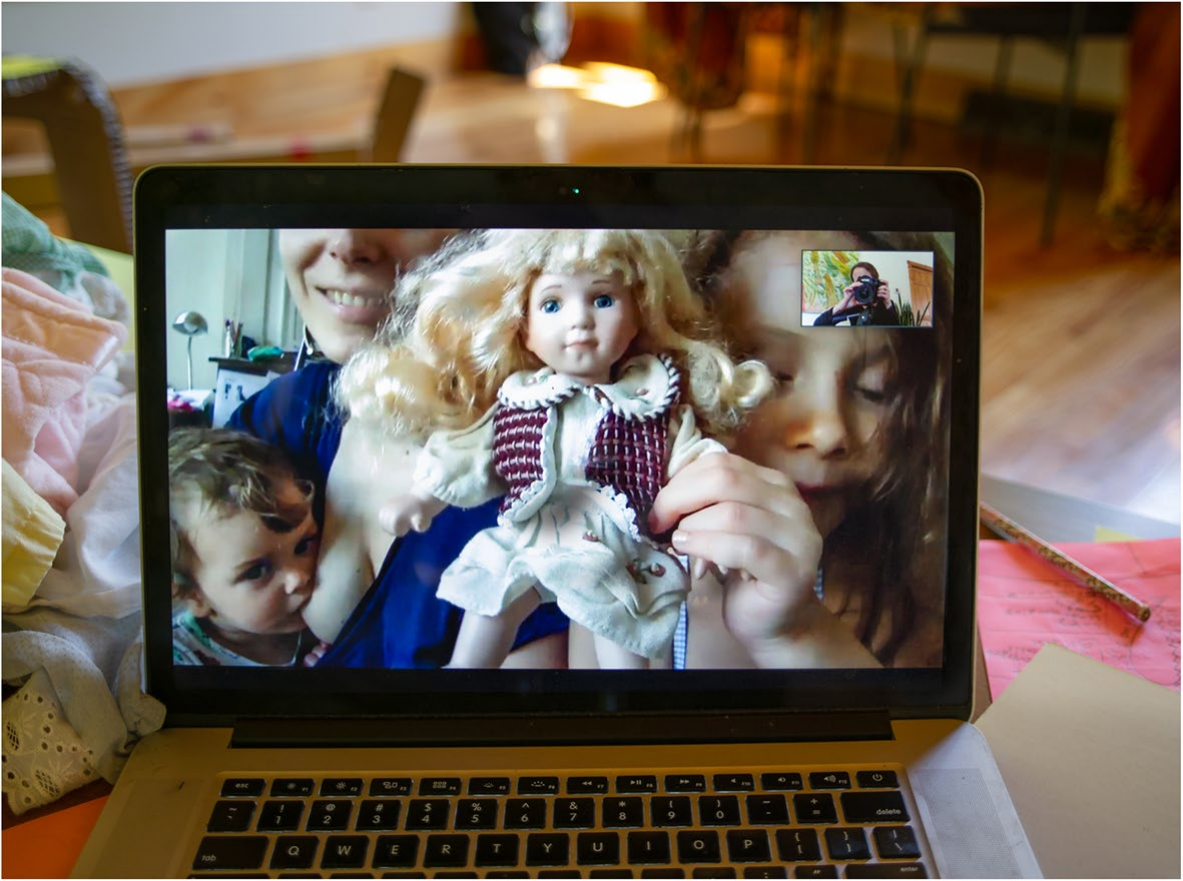
Mathilde Cohen

The peculiar context of the pandemic brought unprecedented emotional involvement and reflexivity in the data collection process [26, 27]. To facilitate scheduling and avoid burdening already overtaxed and time-constrained individuals, we specified that children and other household members were welcome to join the interviews and photo shoots. Their presence or irruptions helped break the ice, creating the feeling of a casual chat rather than the traditional positivist in-depth semi-structured interview shaped by the notion that personal involvement biases the research and/or contaminates the results. Moreover, as a working parent of two who was breastfeeding a toddler (Fig. 1), Cohen was both an outsider and insider to the topic. To counter the lack of physical connection imparted by the remote interview format, she used self-disclosure of her own identity to encourage participants to reciprocate [28]. She often ended up sharing with participants “confessional tales” [29]—that is, her own perspective on lactation during the pandemic—and was regularly offered—or asked for—advice on lactation. On a couple of occasions, Cohen’s children irrupted into the room where she was conducting the interview, becoming part of the conversation, interacting with the interviewee’s children, and strengthening the bonding between researcher and participant. Despite some suggestions that it may be more challenging to establish a sense of connection using online platforms when compared to face-to-face meetings, she felt that the remote experience facilitated rapport building [30].

**Fig. 2:**
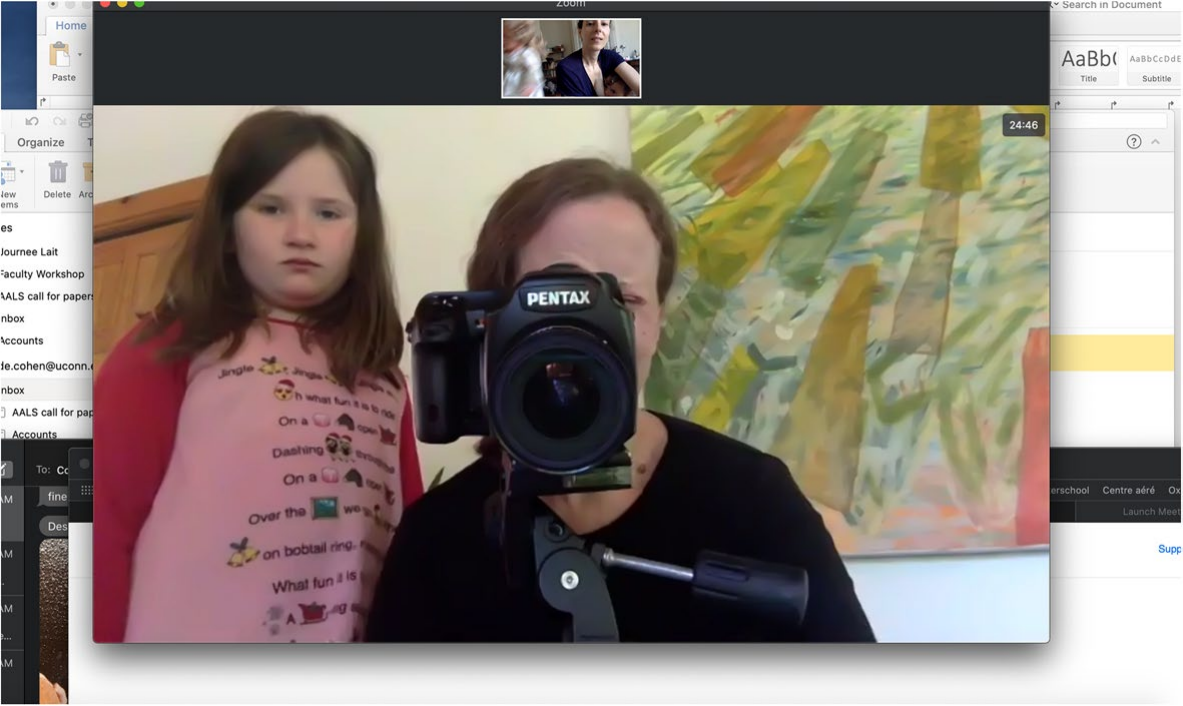
Corinne Botz and her daughter

For visual artist Corinne Botz (Fig. 2), seen here with her daughter standing beside her taking a remote picture of Cohen nursing, online photographing was unprecedented. Botz held virtual sessions using her camera to photograph her laptop through which she connected to participants. In a traditional in-person photo session, Botz would have met the subjects on their front door, developing a rapport as they were walking inside, chatting, and setting up. The remote format made the connection abrupt. Photographer and participants found themselves face-to-face right away without the opportunity to get acquainted, as appointments were scheduled around parents’ baby feeding schedules. These constraints led Botz to feel at times as if she was peeking through a webcam streaming in real time rather than leading a photo shoot.

At the same time, remote photographing may have facilitated collaborative visual representation, whereby the photographer and subject consciously work together to produce “ways of seeing, knowing and showing” that are negotiated outcomes [22]. Participants became partners in the production of the photographs. They used their smart phones’ camera function to grant Botz virtual access to their environments, positioning their devices in different locations and angles or asking relatives or friends to hold them. Botz took photographs of her computer screen with a camera mounted on a tripod, introducing elements of a traditional photo shoot and removing the images from their screen-based origin. The process meant relinquishing some measure of control over the lighting, backdrop, and framing, but it gained in collaborativeness as subjects, family members, and in one case, a child care worker, helped find interesting angles and added unexpected moments in the session. In sum, the move to online qualitative data collection and photography offered an opportunity to decenter the researcher and photographer as the experts in favor of the knowledge and perspective of participants who were invited to tell us about and show us their worlds.

This study has several limitations. First, the sample is small, which does not allow for generalizations. Second, the overwhelming majority of the research participants are middle to upper-middle class. We did interview a mother who was a recipient of The Special Supplemental Nutrition Program for Women, Infants, and Children (WIC), a program that provides foods, healthcare referral, and nutritional education for low-income pregnant and postpartum women and their children as well as a WIC peer lactation counselor (a former WIC recipient herself, as required by the WIC program). But we were unsuccessful at recruiting additional WIC recipients despite setting up a recruitment plan at a WIC lactation clinic. On one level, this failure exposes the shortcomings of remote qualitative data collection—we may have been more successful had we attended clinics and support groups in person to recruit participants [31, 32]. On another level, it reflects the very challenges of lactation in quarantine the article aims to expose as lactation support programs transitioned to virtual environments. Most importantly, it is already hard enough for under-resourced parents to give birth and care for infants during the crisis. They may not have the time, mental energy, and desire to connect with unknown, race and class-privileged researchers and photographers to share their infant feeding experience.

## Results

We identified four main themes in participants’ narratives about how they experienced and made sense of human milk feeding during the pandemic. First, participants conveyed the loneliness of early parenting and breastfeeding in social isolation using the trope “It takes a village to raise a child.” Second, they approached human milk feeding as a coping mechanism with the crisis. Third, the (in)visibility of lactation within the context of their heightened multitasking appeared as a significant concern. Finally, human milk was socially constructed as a relational link during a time of unprecedented isolation.

### Social Isolation—“It takes a village”

For our research participants, the pandemic transformed lactation, a practice that ordinarily thrives with physical closeness and social support, into a solitary experience, from postpartum lactation initiation to breastfeeding toddlers.

The various lactation specialists we interviewed pointed out that the management of the crisis, especially during the first months, led to harmful separation strategies between parents and infants, families and their support system. The Director of a Neonatal Intensive Care Unit (NICU) in the Northeast, Catherine, a White neonatologist, deplored that at her NICU, COVID-positive parents and those who live with them were initially prohibited from visiting their premature babies for 14 days, making the establishment of lactation extremely difficult.

For some households, however, the social isolation resulting from the response to the pandemic helped. Catherine noticed that breastfeeding rates at her hospital rose by 10% in April and May of 2020. She explains,

Women are admitted, and they have their baby, and maybe they’re nervous, and they don’t want their baby to go to the well-baby nursery, so they end up just nursing the baby all night long. They have no visitors, so Uncle George and Grandpa Jim and their well-meaning neighbor are no longer coming to see them… Nobody is saying, “Oh, I want to hold this baby.” Normal processes of moms doing skin-to-skin and bonding with the baby can happen. There’s no interruptions anymore.

But limits on the number of visitors can have a negative impact on lactation, other lactation professionals pointed out, as they may prevent key relatives or friends from helping. Additionally, some participants highlighted that early hospital discharges, which became common, may have contributed to inflating breastfeeding rates in hospitals. Parents were discharged after having been counted as breastfeeding, but before their milk had the time to replace colostrum, thus before most lactation difficulties leading to weaning arise.

**Fig. 3:**
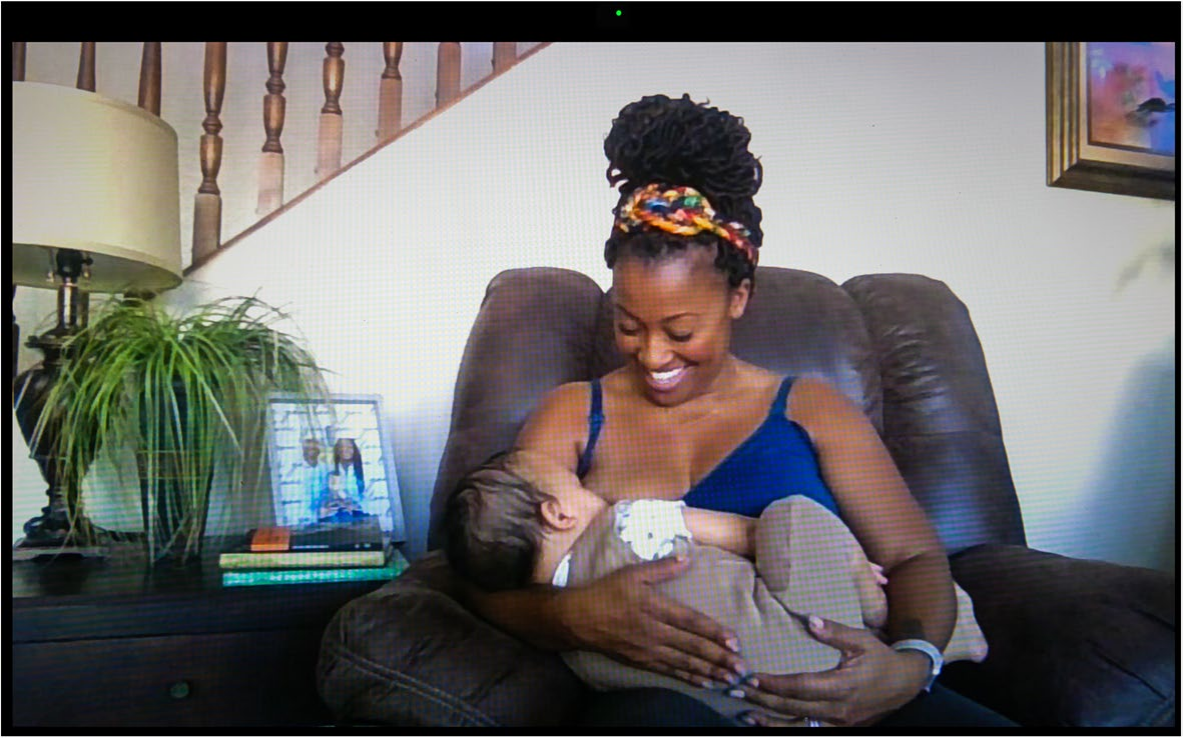
Bethany

Once back home from the hospital, the new parents we interviewed felt extremely isolated. Bethany (Fig. 3), a first-time Black mother and lawyer who gave birth at the beginning of the pandemic, commented, “if I had to use one word to describe that experience it would be lonely. Very, very lonely.” Despite her loneliness and the ordeal she went through to establish lactation without access to in-person lactation support, breastfeeding is a source of intense joy and pride for her—in Botz’s picture she is beaming while feeding her baby. Indeed, isolation can be a blessing or a curse depending on the situation. If some parents missed the emotional and material support friends and relatives would have normally contributed, they were also shielded from the intrusion of outsiders, their judgments, and their gaze. Seclusion allowed a few to express milk or feed at the breast when and where they wanted without disruptive interventions or having to cover up. Anahita, a Black attorney, confided, “one of the unexpected privileges of the pandemic is that there aren’t people coming by.... I can just freely breastfeed in my house and not feel shy about that.” Carla, a Sisseton-Wahpeton lactation consultant and founder of several initiatives to support breastfeeding in Native American communities, observed that the Indigenous families she serves are disconnected from their elders, which is a huge loss, but “at the same time, people don’t have doctors undermining their experience, so they’re really successful in breastfeeding because they don’t have someone that’s constantly checking their babies’ weight and freaking them out about their supply or their babies not getting enough milk.”

**Fig. 4:**
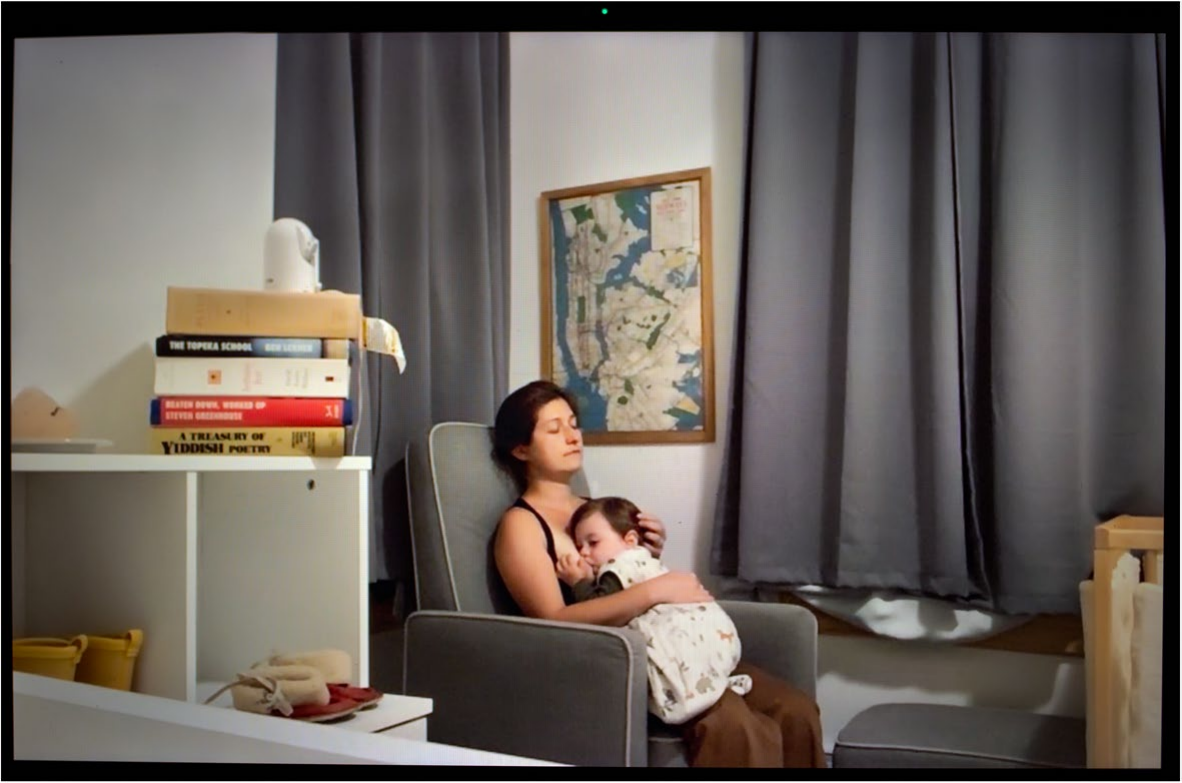
Nina

The outbreak concurrently diminished the lactation counseling offering by limiting in-person consultations and broadened it with the turn to online spaces. Black breastfeeding advocate Nicole saw a reduction in cost for online lactation counseling, including sliding scale options, suggested donation, and free help. But as White nurse practitioner and lactation consultant Judith remarked, providing remote services “is not easy. When a woman is having difficulties with latching and she’s using a phone, holding it over her shoulder, and I’m talking her through how to latch, it could be difficult. I’ve had two patients turn around and say, ‘I can’t do this,’ and—and, ‘I quit.’... One woman gave up entirely, and one woman said, ‘I’m just going to pump.’” Similarly, Nina, a White midwife, noted,

online resources are better than nothing, certainly. But I just don’t think there’s any way that it’s really going to replace the feeling of getting out of your house and going and being physically with other humans going through the same thing that you’re going through. . . . Finally, now at least there are lactation counselors and postpartum doulas who go in person to make house calls and thank God. Because, you know, for a while, nobody was going. (Fig. 4)

As a mother of a baby herself, she was also speaking from experience. In the picture, she is nursing her son to sleep, while trying to enjoy some rest. Alicia, a White lactation consultant who directs a WIC lactation clinic serving impoverished, primarily refugee and undocumented immigrant populations, noticed that “a lot more people are willing to give breastfeeding a try because they’re home with their babies,” before adding that “there’s more stress involved because they don’t have as much access to support.”

**Fig. 5:**
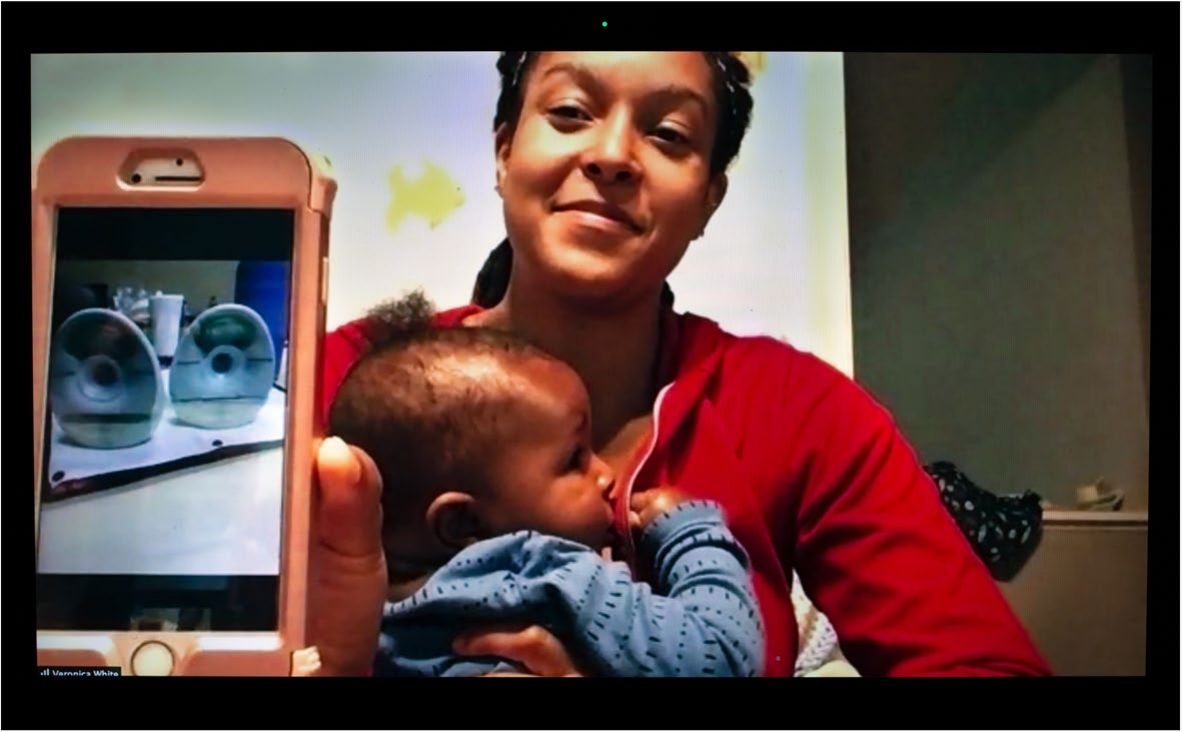
Victoria

**Fig. 6:**
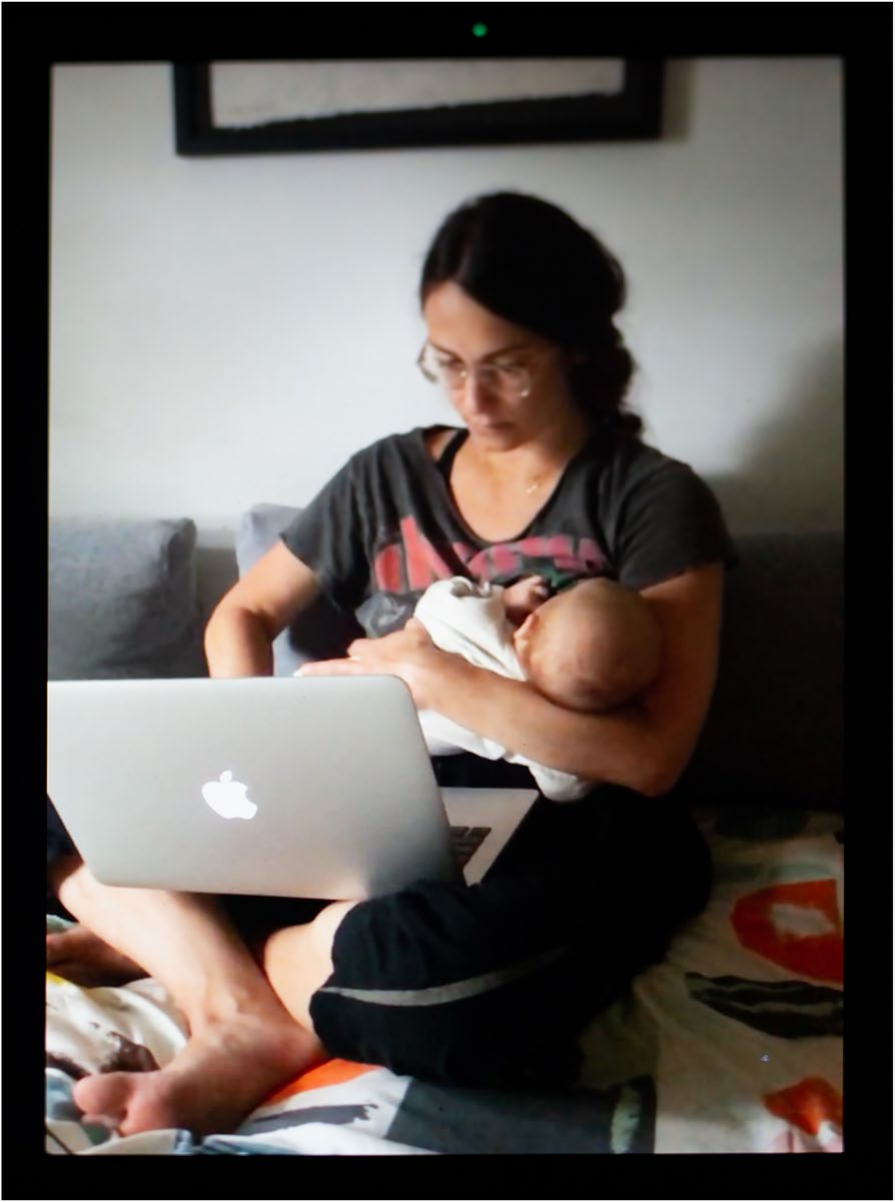
Yael

**Fig. 7:**
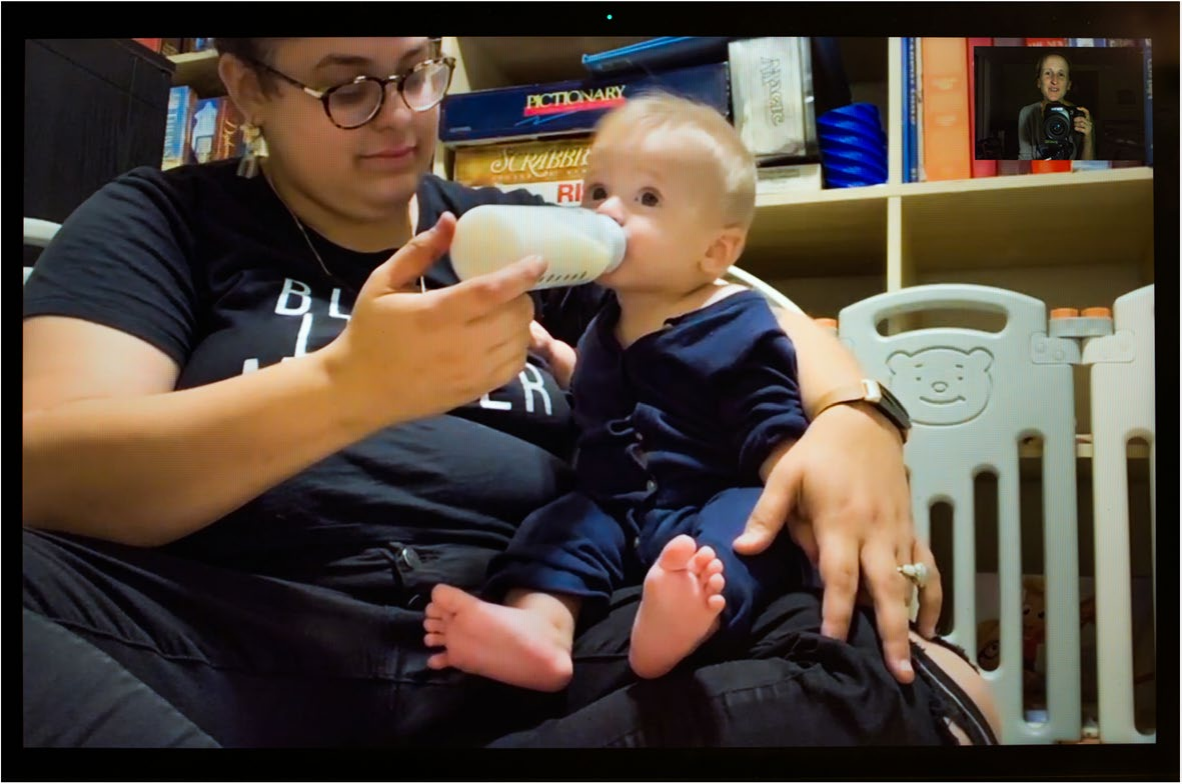
Aimee

Again and again, the parents we talked to cited the proverb “It takes a village to raise a child” to characterize the primary loss they experienced—the sudden and brutal cutting off from their social circle and various support systems. Black critical care nurse Victoria (Fig. 5), who gave birth to her second child in the first weeks of the pandemic, found the postpartum experience radically different than with her first child, “with my daughter, I was part of different mom groups, and I had playdates, and I could go for walks and go the park with different people, and with him... I had to do everything over Zoom, and it’s different than when someone’s physically there with you... it’s more lonely this time.” Similarly, Yael (Fig. 6), a White entrepreneur who gave birth to a second child early in the pandemic exclaimed, “so much of early motherhood is about commiseration with your mom friends. I miss it terribly. I feel that my mom friends are the ones that lifted me up and kept me alive, literally.” In this picture, she is seen breastfeeding her newborn at her parents’ suburban house, where she temporarily relocated, catching up on work for her online business. Queer Afro-Latina new mom and community organizer Aimee (Fig. 7) explained, “the main issue is a lack of community support for women. The majority of people supporting women are other women and that’s how it’s been the same since the beginning of times.”

**Fig. 8:**
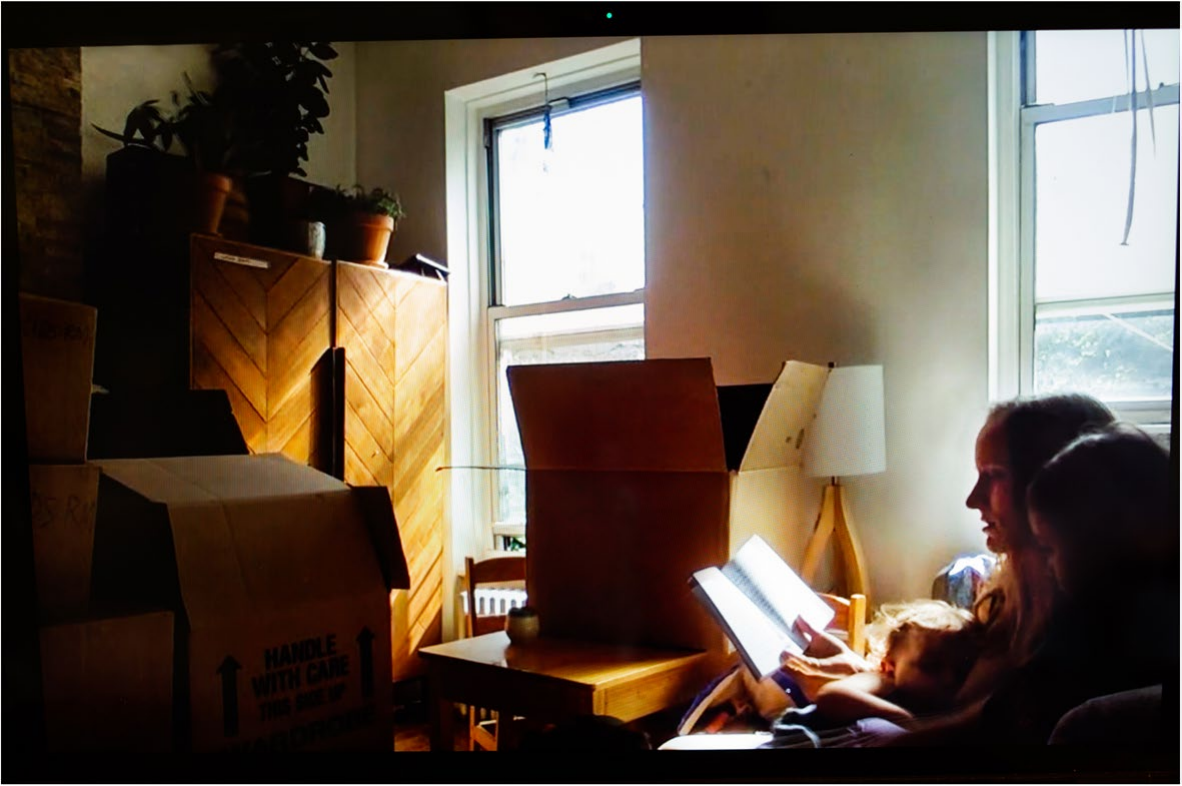
Sienna

**Fig. 9:**
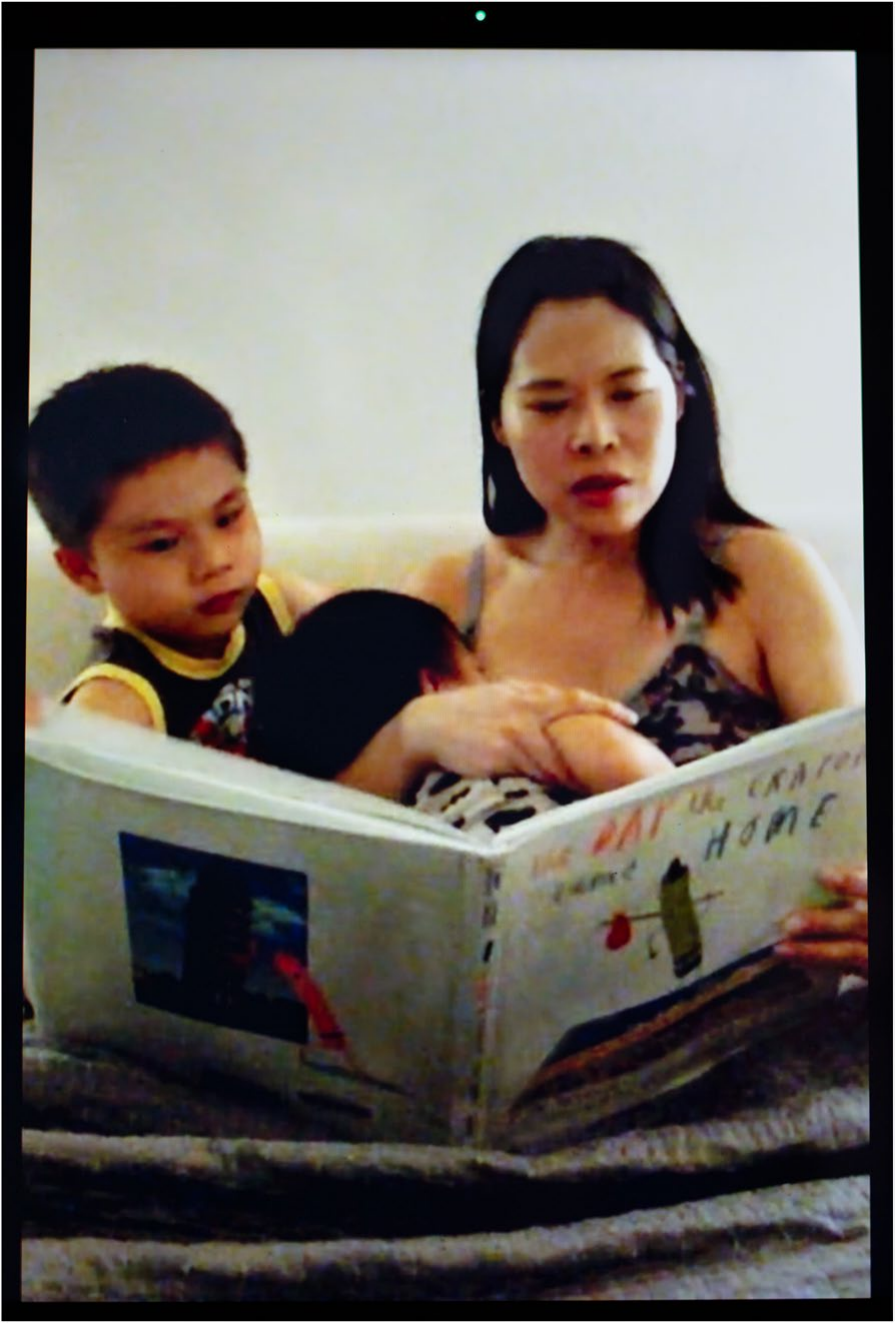
Li-jing

While caring for infants is extremely demanding physically, cognitively, and emotionally, social isolation creates different hardships for different people. The more privileged, who live in single-family homes or apartments, talked about the fatigue of being one-on-one with their children for extended periods of time, while the least resourced, sharing homes or rooms with multiple people, regretted the lack of privacy. For affluent parents, the crisis was an opportunity—or catalyst—for relocating from urban areas to the suburbs, the country side, or foreign countries. Sienna (Fig. 8), a White bar owner who had been “trying to figure out how to get our kids a backyard and have a little more space for a while,” moved out of the large city where she resided when her bar was “shut down.” In this photograph, she can be seen reading stories to her children while nursing in her city apartment filled up with cardboard boxes in preparation for the move. Li-jing (Fig. 9), a Chinese-American journalist and breastfeeding activist, recounted that “the wealthiest families in the Taiwanese community [in her California town]—the mothers—took the kids and flew back to Taiwan while fathers stayed here to work. They feel that the Taiwanese government is doing a much better job to prevent the spread.”

**Fig. 10:**
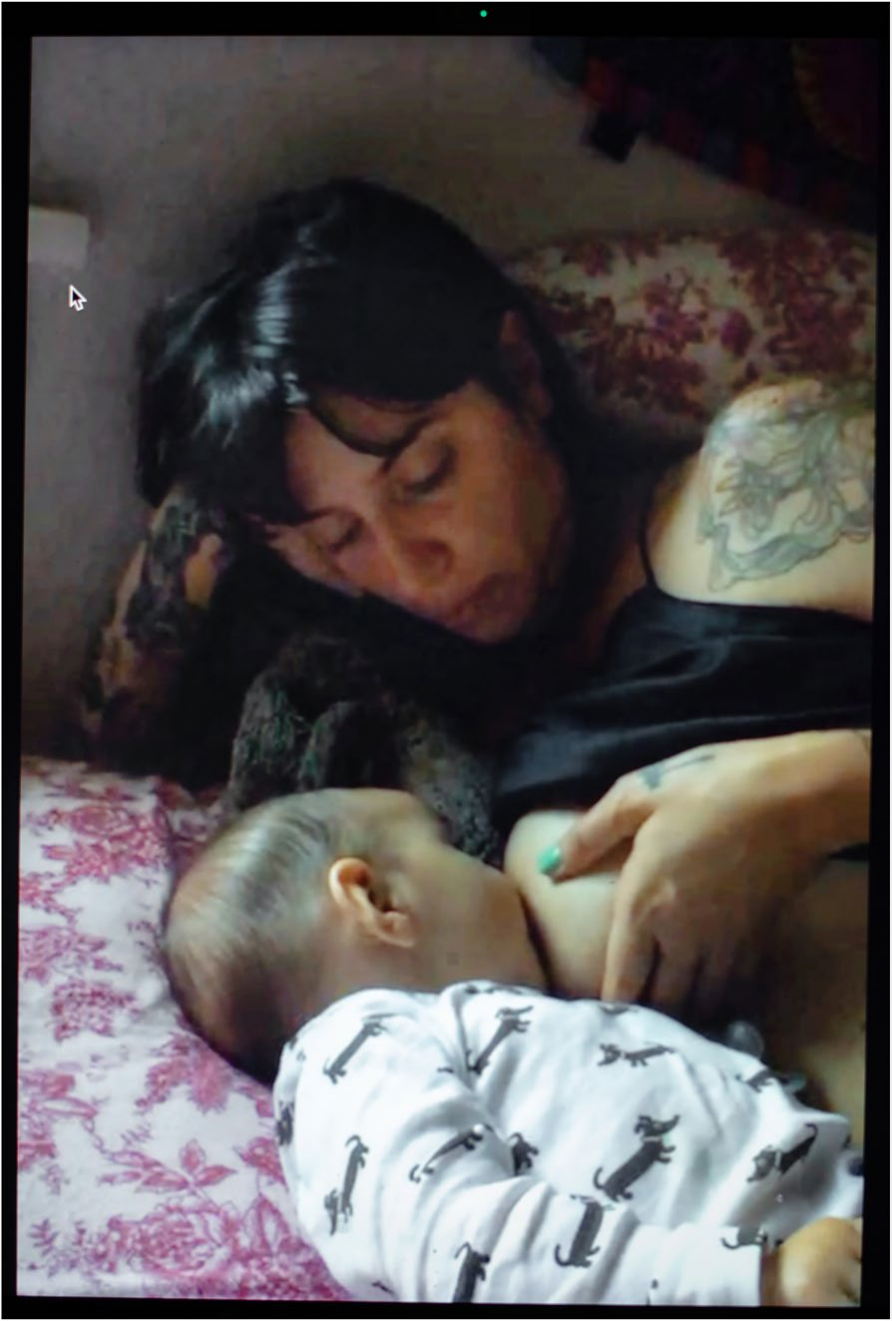
Theadora

For less-resourced families, stay-at-home orders were a source of intense psychological and spiritual anguish. Latina tattoo artist Theadora (Fig. 10) gave birth a few days before the March 2020 stay-at-home order went into effect in her state. She was expecting her mother to move in with her to help with the baby, but ended up alone with her husband, ‘You need a village to raise a baby—it’s true 100 percent, and I didn’t know how much until you don’t have that village.’” It all came to a head when she was hospitalized for a breast infection and could no longer breastfeed nor hold her baby due to her debilitating pain and drug regimen. In this picture, she wanted to be represented lying down, feeding at the breast serenely, after recovering from her illness and being able to nurse again painlessly. Natasha, a Latina WIC recipient who immigrated to the United States a couple of years before the outbreak, emphasized that she had to do “everything alone” upon returning home from her Cesarean section. Her main connection to the outside world used to be through her church and outdoor play with her kids. She bemoaned that she “cannot do that” anymore. “That’s heartbreaking,” she added—life has become “very monotonous, because it’s every day the routine, the stay at home.”

**Fig. 11:**
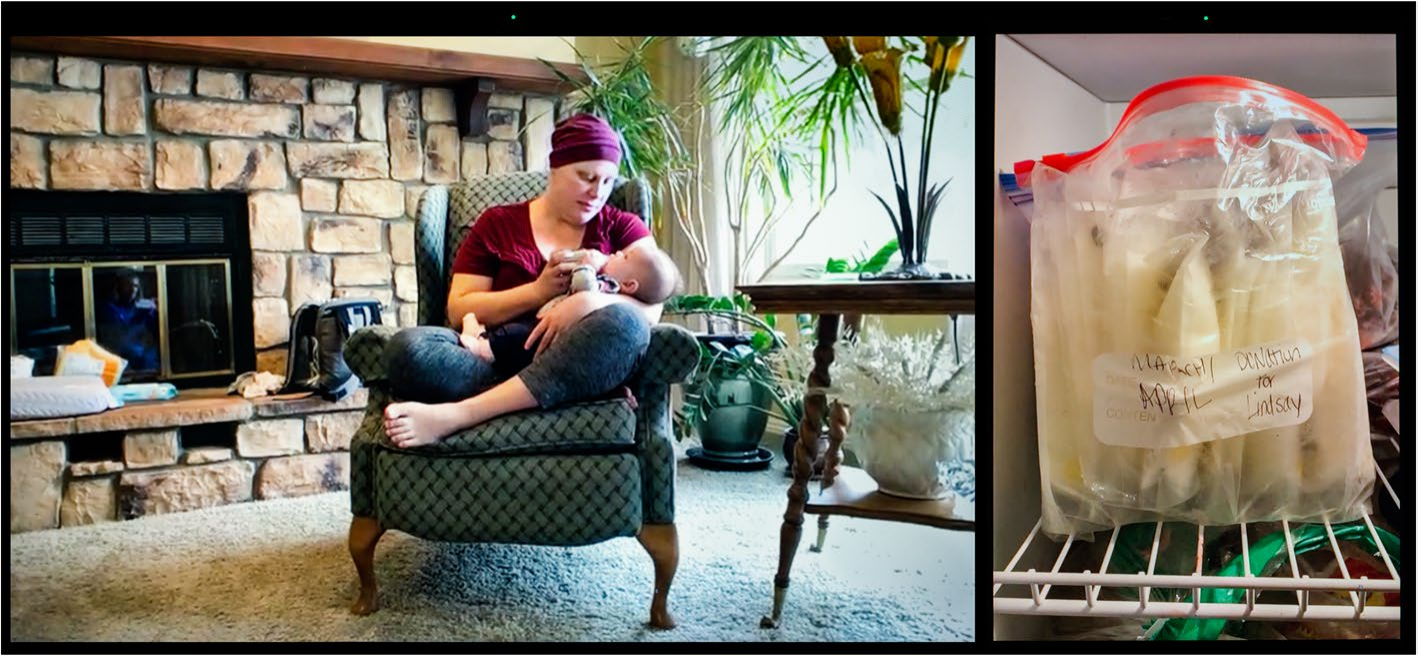
Chelsay

Isolation can be particularly challenging in certain life situations and for certain communities. Under the shelter-in-place orders in her state in 2020, Chelsay (Fig. 11), a White nurse, found herself going through chemotherapy while caring for a 4-year old and a baby born with a genetic disorder of bone growth, while her husband worked remotely and faced significant immigration issues. She stresses that “one of the things that I was so frustrated and just really angry about when the stay-at-home order came was that it ripped a lot of my support system away.” She was suddenly cut off from her “very strong village of mamas.” As someone who used to spend significant amounts of time parenting outdoors in the company of others, she resented being stuck indoors in an apartment not “know[ing] how to be a mom at home.”

Carla emphasized the immense intergenerational and spiritual costs of COVID-19:

for most of our tribal communities. The postpartum period—those first 40 days after a baby is born are—is sacred. There are ceremonies. This is a time when the baby would be cared for by the mom who would be cared for by her mom and her aunties and her grandma, and people would be cleaning and cooking and just loving on them so that they can just focus on nursing and getting to know each other, and families do not have that.

Given the health disparities affecting Native-American communities as evidenced by the skyrocketing per capita coronavirus infection rate in the Navajo Nation during the spring of 2020 [6], Carla notes that people are “afraid to have elders come into their homes; they’re afraid of exposing them to the virus.... but because of that they’re missing out on the teachings.” For the next year, or possibly longer, she laments that critical rituals such as sweat lodges, pow wows, canoe journeys, and the honoring of the newborns will not take place, with “devastating” consequences given “the importance of ceremony and culture and the reclaiming of cultural practices for health and wellbeing. For a lot of people ceremonies is what keeps them sober. It’s what keeps them alive because they’re part of a family that’s healthy and takes care of them. And how are we doing that during this time?”.

### Milk as a coping strategy—“the milk... is gold for her. She’s gonna get antibodies”

The second theme we identified is the construction of human milk feeding as doubly necessary during the crisis because of its protective effects against COVID-19 and many parents’ near-constant proximity to their children. Human milk became a means to cope with the health and social effects of the crisis: the risks of falling ill to COVID-19 and the intense stress of being isolated without support.

Breastfeeding and human milk are glorified in US society as “‘liquid gold,’ believed to provide immunological benefits unparalleled by infant formula” [33]. The initial uncertainty as to whether SARS-CoV-2 could be transmitted via bodily fluids, coupled with the politicization of the health crisis destabilized this social construction of human milk for some, while reinforcing it for others. Li-jing (Fig. 9) says that many new mothers in her community chose not to breastfeed: “They speak limited English, and they still rely on information from their home country—say, from China, from Taiwan, or from the Philippines and since the outbreak, the Taiwanese and the Chinese governments have been telling moms to switch to formula.” It is also a “trust issue,” stemming from the anti-Asian coronavirus rhetoric encouraged by former President Trump, which is “failing Chinese-American moms. They don’t trust the American CDC as much as before.” Li-jing continues, “we already know that second- or third-generation mothers breastfeed a lot, but it’s the first-generation moms; they don’t. It’s harder for them to get started. They don’t get the support they need in the hospital” in part because of the dearth of lactation consultants who identify as Asian or speak their languages [34]. With the crisis, these parents found themselves more than just physically, socially, linguistically, and culturally isolated—according to Li-jing, they felt less comfortable seeking and accepting support from outside their community given the surge in anti-Asian violence as well as anti-mask sentiment.

Nearly all the parents in our sample expressed that the pandemic changed their infant feeding patterns and goals. Several cited the potential immunological benefits of human milk against the coronavirus as a reason for not weaning and even for relactating. Carla reported hearing from the communities she works with “lots of people wishing they were breastfeeding” to provide their children with antibodies. She added that “the number of people who reach out to us for inducing lactation, asking about relactation, or upping milk supply has gone up.” For Chelsay (Fig. 11), a perinatal nurse, COVID-19 was an additional reason to feed her infant human milk. She discovered that she had breast cancer when she was 35 weeks pregnant, just a few weeks before COVID-19 exploded in the United States. Of her diagnosis, “the most devastating part,” she says, was that she “wouldn’t be able to do that [breastfeed] for her” daughter. Her oncologist gave her “three weeks and a day to nurse her” baby, after which she had to wean abruptly to begin treatment. She found mothers in her community who gave her some of their extra milk. On this image, she is seen in her living room feeding her baby donor human milk. Both she and her daughter are immunocompromised, which Chelsay explained made human milk feeding even more critical—“she’s [her daughter] getting antibodies from all of these moms and building up this great immunity.” As a health worker working on becoming a certified lactation consultant, Chelsay knew that human milk was not a vector of SARS-CoV-2 contamination. She even hoped that one of the batches of milk she received was from a COVID-positive donor—“that milk might be extra special,” as it could include SARS-CoV-2-specific antibodies.

Human milk feeding was not just a way for parents to protect their children from the virus. Several also experienced it as a form of coping mechanism when they were isolated from their support system and yet in near-constant contact with their children. This increased proximity intensified lactation. In many households, there were no more schedules, no more breaks, no more boundaries. Several women indicated that their baby or toddler sought nursing at breast nearly constantly. Midwife Nina (Fig. 4) declared enjoying being home more than usual with her continually nursing toddler, but “then sometimes I just feel like, ‘Just get your hands off me, please.’”.

Before the virus hit, a number of research participants had planned to wean or increase their milk expression regimen to gain more autonomy. They reversed course. The decision was not only motivated by the desire to “protect” their children from infection. It was also a response to the continual closeness to their children. Li-jing (Fig. 9) says of her toddler, “I think this pandemic has prolonged our breastfeeding plan because he has been home with me, and he became very attached to me, and it’s very hard to wean him in this situation.” She chose to be photographed during her children’s bedtime routine. She is seen reading a book to her oldest son, while nursing her youngest.

**Fig. 12:**
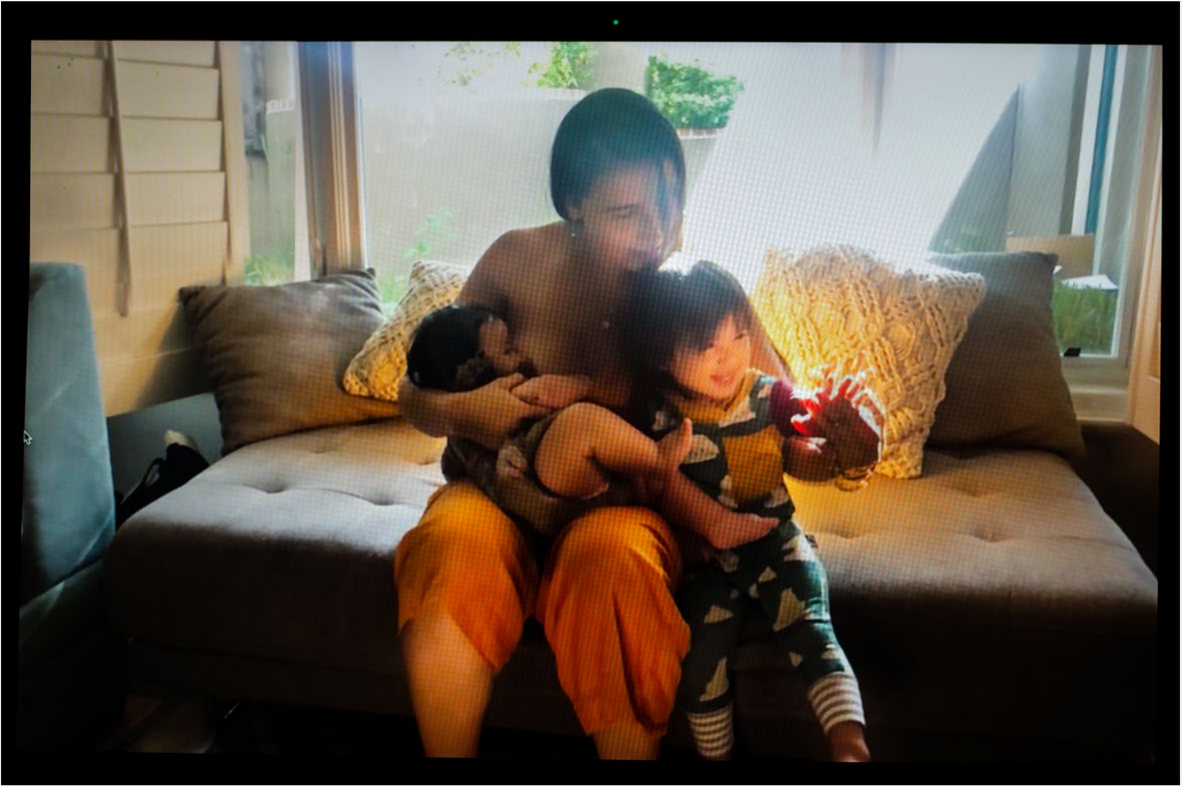
Vera

In early March 2020, stay-at-home mother Vera (Fig. 12) was prepared to pump so as to leave the house from time to time, allowing others to feed her baby, “but now it just sounds like a horrible idea.” She let go of her nanny; her niece, who used to live with her and help out, left. She’s alone all day with her kids while her husband works long hours from their basement. In the picture, she nurses her baby, while playing with the sunlight with her toddler. These two images—Li-jing’s and Vera’s—illustrate how the pandemic affected the meaning and practice of lactation, but also how, more than ever, lactation has become a task to combine with many other paid and unpaid ones, such as here caring for older children.

### Multitasking—“I have to breastfeed while I’m working”

Nearly all the parents we interviewed expressed their frustration with the need to combine lactation with a host of other paid and unpaid forms of labor. Essential workers talked about having to express milk at work using heighted precautions to avoid contamination with SARS-CoV-2, which they feared could be present on hands and surfaces, while holding onto jobs that often failed to provide them with adequate lactation breaks and facilities. Even for Nina (Fig. 4), who enjoys a favorable social and professional position as an independent midwife, and access to her hospital’s on-call room and hospital-grade pump, the decision and logistics around whether and how to express milk was fraught, “I went back and forth a lot in the beginning about should I even be pumping at all when I’m at the hospital? Is that a safe thing to do, you know, in terms of COVID prevention? Not pumping at all was not an option because for my own physical well-being I needed to pump, but then, should I toss that milk and not save it? Is it not safe?” Victoria (Fig. 5) went back to working 12-h shifts as an ICU nurse in June 2020 after giving birth to her second child in April. She was only able to maintain her lactation thanks to the $500 high-tech, wearable pump she purchased out-of-pocket, as it was not covered by health insurance,

because of the pandemic and everything, we’re really short-staffed, so there is no way I could get away four times during my shift and pump. With my Spectra [a regular electric pump], I usually pump 15-20 minutes, but . . . we only have one lactation room for the whole hospital, so I have to go down to the third floor, set all my stuff up, then actually pump, then pack up all my stuff, take it back to my unit, clean everything—it would take, easily, 30 minutes, and I can’t do that four times per shift.

In Botz’ photograph, Victoria is seen at home feeding her baby at the breast and showing a picture of her wearable breast pump on her phone. This pump allows her to express milk while caring for patients unbeknownst to others. While pumping, she can empty catheters, push stretchers, pull up, turn, and transport patients, and take them to get MRIs or CT scans. Other than bending over, which could spill the milk, when she is wearing the pump “there’s nothing I can’t really do, so, it allows me a lot of freedom.” Lactation is still “a lot of work” she emphasizes, before expanding, “the whole pumping thing is a lot of work, and trying to figure out the schedule and—if you do have a regular pump break—or even if like me you are using the wearable pump, you still need a supportive environment.”

**Fig. 13:**
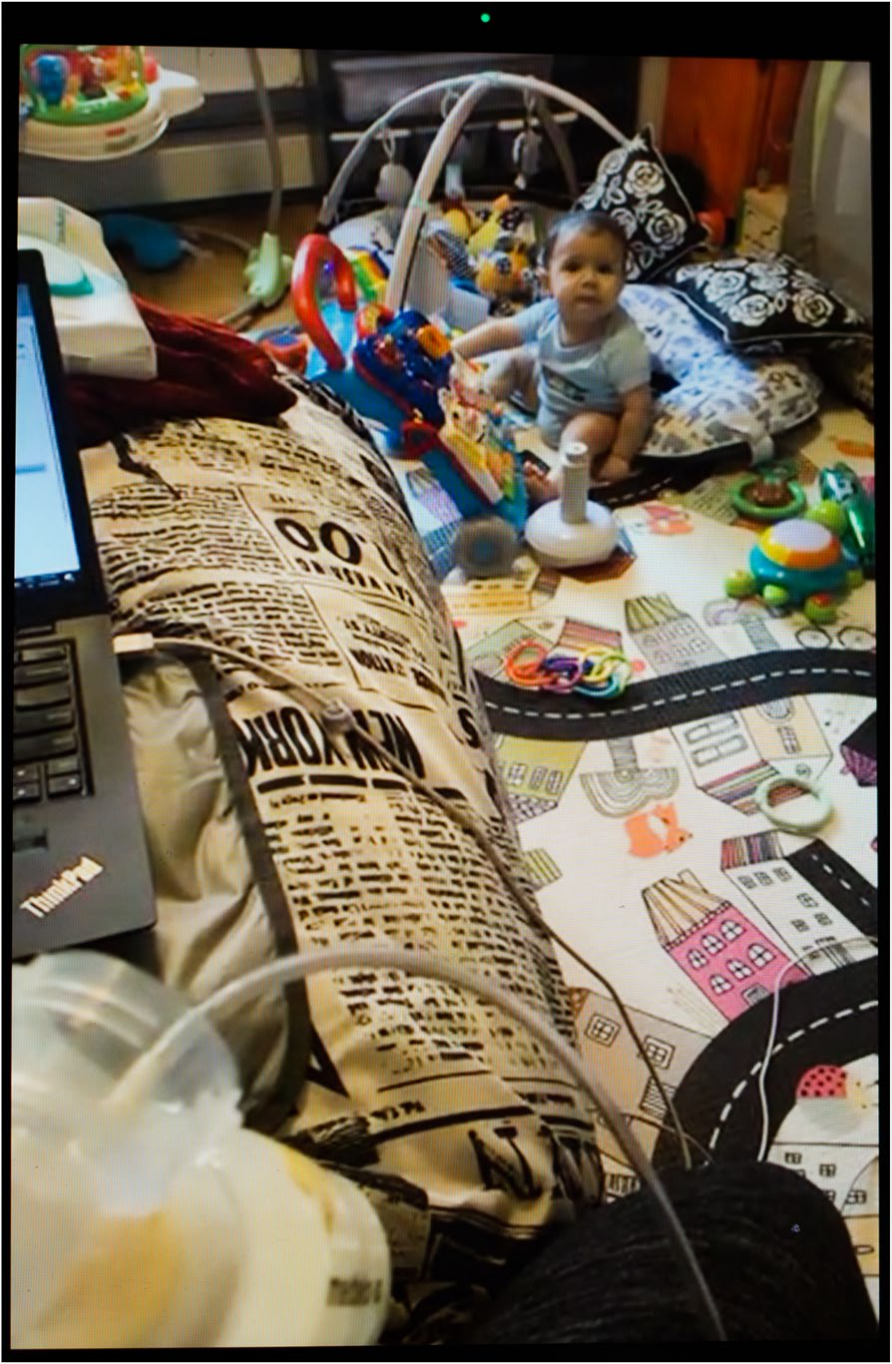
Iris

For teleworking parents, being home was both a benefit and a burden depending on their professional and life circumstances. White-collar jobs with good salary and benefit protections afforded parents the financial ability to secure in-home childcare or at least the flexibility to work around their lactation schedule, rather than the other way around. For instance, Latina neuroscientist and single mother of two Iris (Fig. 13) is an exclusive pumper—she only feeds her infant expressed human milk with no suckling at the breast. In the photograph, she is expressing milk while catching up on emails and keeping an eye on her baby who is looking at her from his play gym. As she notes, “the only advantage [of the pandemic] is that, since I am working from home, and I am at home, I sit next to the machine [the breast pump] all day and all I have to do is run to the kitchen and get my stuff and just strap it on.” When her nanny watches her children, she can pump through meetings and classes from the comfort of her apartment, rather than juggle the complicated logistics of pumping at work.

Brielle, a Black certified breastfeeding specialist and teleworking mother heard multiple reports from her own practice and the online support groups she moderates that many low-income women were forced to give up breastfeeding due to the difficulty of obtaining adequate support and the overwhelm caused by the loss of jobs, health insurance, and childcare. One of her clients told her, “I can’t even nurse the little one anymore because I have the three older ones all over me all day, and I never get a moment.” Another confided, “Listen, you know, I’m getting WIC. I’m getting food stamps. It’s a little bit easier to just order and pick up the formula, but, you know, I’m out here still trying—in the middle of a global pandemic—to find a job with a six-week-old. It’s just not worth it, you know [to attempt to breastfeed].” Still another stated, “I’m home with all four of my children now. You know, I don’t get pump breaks.” Brielle commented, “you have to respect that you don’t know what she’s going through. Breastfeeding works when it works, but when it doesn’t work for a family, it sometimes can be more of a burden.”

**Fig. 14:**
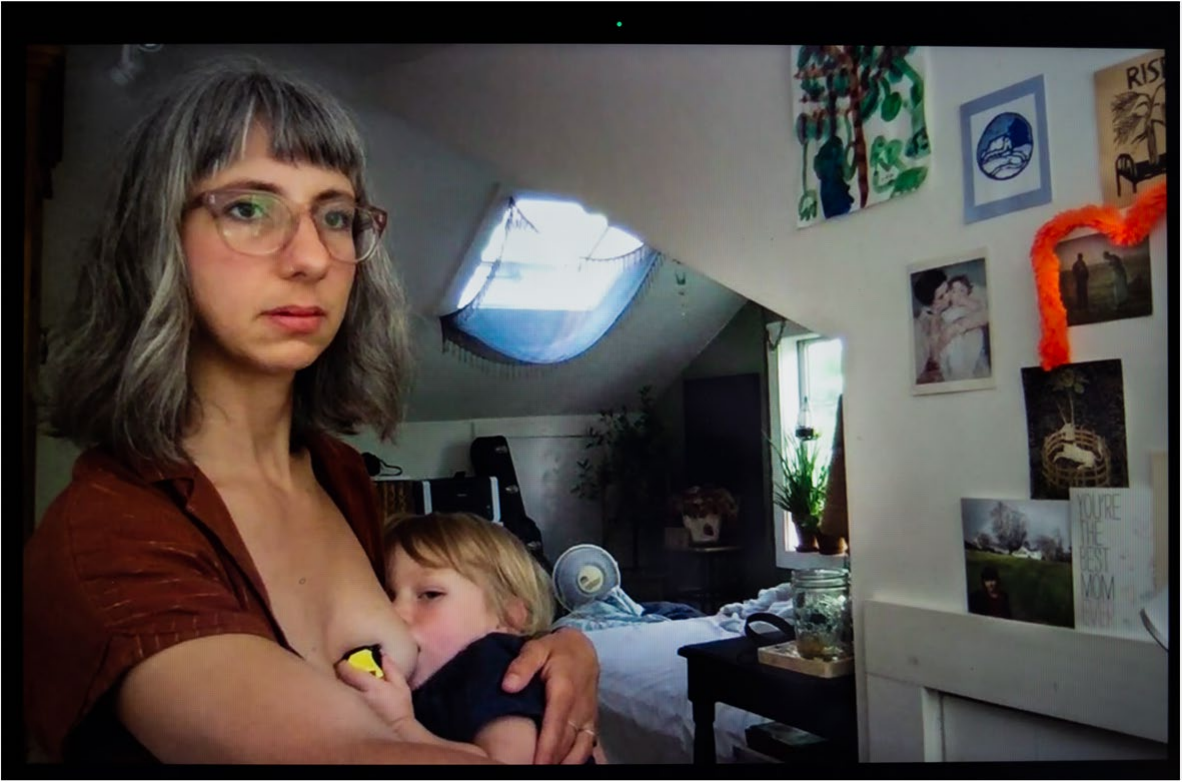
Talulah

If multitasking is arguably an essential part of parenting, the pandemic has brought it to a new level. In many instances, lactation became an extra burden in addition to wage work, housework, homeschooling, caregiving of children and adults, and admin, among other tasks. But lactation could also be a pleasure and an asset when putting a child at the breast soothes them, keeping them quiet and happy while their parent could complete a work task, a conference call, or interact with another child or relative. Stay-at-home orders saved Talulah and Valentina, both White and married, time and money by relieving them of their commutes to city jobs. On the downside, their children’s daycares and schools closed, necessitating constant juggling so as to combine breastfeeding with other responsibilities. Talulah (Fig. 14), a photographer, is seen editing pictures while nursing her toddler. She shares the childcare with her husband, who also works from home. But unlike her, he does not lactate on top of working, “we divide the days in half, and that, of course, always means that I have to breastfeed while I’m working.”

**Fig. 15:**
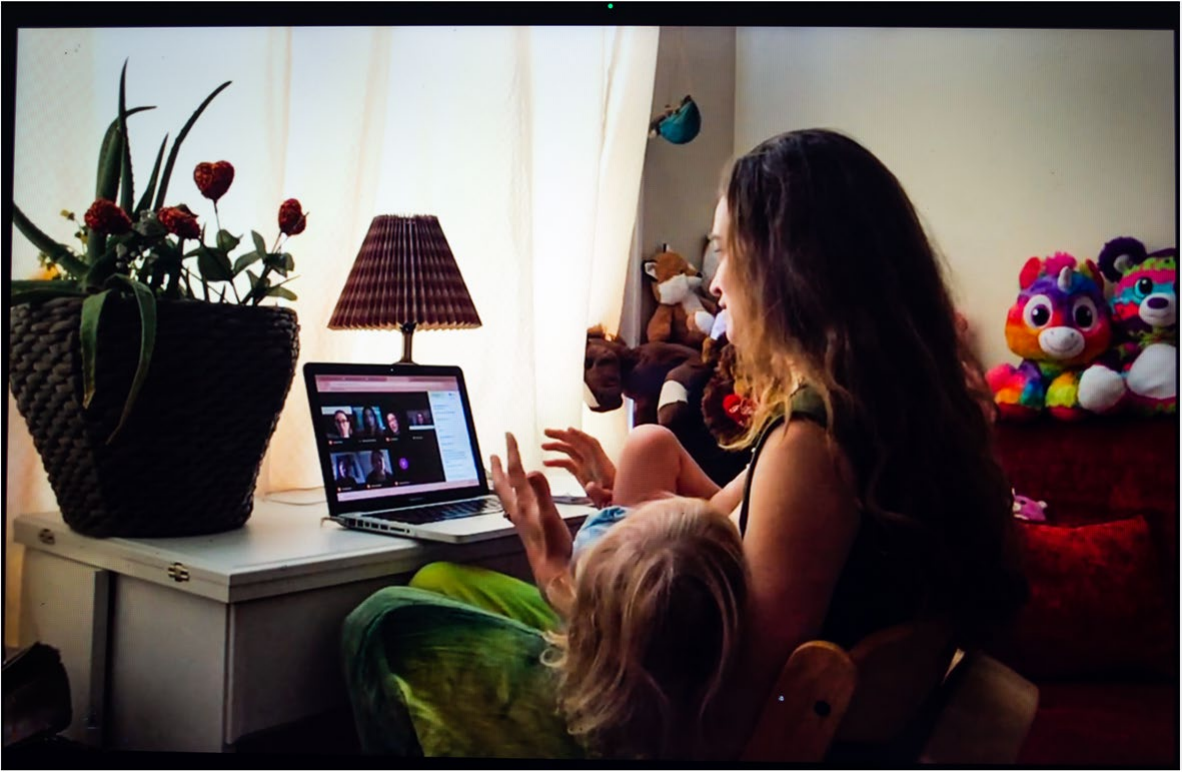
Valentina

High school math teacher Valentina (Fig. 15) shared the difficulty of teaching back-to-back classes and attending online student and faculty meetings while home alone with her toddler and two older kids. Her husband works outside the home trying to keep his restaurant business afloat. She was photographed seated in a makeshift home office in a corner of her living room while participating in a professional video-conference meeting with her toddler on the breast. Given her circumstances, nursing sometimes allows Valentina to be a more engaged worker, as she can simultaneously interact with her various work constituencies and keep her daughter happy:

I’m supposed to be present with you [colleague or student]. She’s [her daughter] on my boob. . . . The baby is often on my lap, and they’ll see—she’s on one, and then I flip her, and they’ll see her flipped to the other. I wasn’t hiding the video…. I kept everything on because it’s easier to talk while nursing than to type while nursing. I would get these text messages when I was done . . . Other nursing moms would notice what was happening, but I’m not sure anybody else did.

For women suddenly home due to the COVID-19 crisis, the inability—or reduced ability—to work could be both enraging and financially difficult.At the same time, it could alleviate the dissonance they frequently feel between the expectation that they be nurturing and present for their children and that they be productive and competitive at work. Talulah thus recounts,

for two or three months, I wasn’t really working that much so I was really with the kids more than I normally am. While there was of course the tension of feeling like I wasn’t accomplishing anything or I wasn’t getting to work or I was just caretaking, there was comfort in that, and it was nice to be able to just be with him [her breastfeeding toddler] and to be available for that rather than that tension that you find as a mom when you’re, like, “Okay, I’m going to drop you off at school and go to work for eight hours, and that’s going to be hard for us emotionally but it’s also going to make me a good mom and make me sane.”

Conversely, especially when their wages and benefits are vital to the family, some women may find relief in having to loosen their parenting standards to get work done. Meals become snacks children fix for themselves. Home schooling is not happening. The home is messy. Videos are the new babysitters. As mother of two Victoria (Fig. 5) avowed, “I lowered a lot of my expectations as far as the things I will be doing around the house. I make my life super simple.... it is impossible to just do everything and watch the two of them all day so I’m just trying to be more realistic.” Mother of three Valentina (Fig. 15) stated, “there’s a lot of screen time.... the baby’s watching TV, which I never, before quarantine, would have allowed.” Li-jing was the only parent to say that her “parenting standards are still the same. I’ve heard many parents who have loosed on screen time, but in our house it’s still the same.”

Several participants thanked us for conducting a research project on lactation, as they felt that this aspect of their unpaid, gendered labor had become even less visible with the pandemic, now that it was mostly confined to the home. For instance, Chelsay (Fig. 11) announced, “I’m so happy that you’re doing this research—that you’re interested because I think it’s this kind of unseen impact on mothers that really reaches right to your soul as a mother, but it doesn’t get talked about.” Lactating mothers yearned for visibility and recognition for what they consider to “a lot of work,” as Victoria repeated several times. At the same time, the condition of confinement, can liberate some parents from some of the force of oppressive norms around nursing in public. For instance, as noted earlier, Anahita felt freer to breastfeed in her own home without fear of censure or worry of offending anyone. Since no one was visiting, she could breastfeed without the unwanted visibility that comes with complex attitudes around lactation.

For other participants, the pandemic and the move to online work and socializing brought about increased visibility to their lactation. Some needed or chose to breastfeed in a new sort of public venue—videoconference meetings. Valentina’s colleagues could now watch on Zoom (if they paid attention) her wage labor redoubling as lactation labor (Fig. 15) as she often fed her daughter at the breast during remote faculty meetings. In ordinary times, this sight would have been unavailable, either because she arranged her lactation schedule around her work hours, or because she expressed milk at school inconspicuously in an enclosed, designated space.

**Fig. 16:**
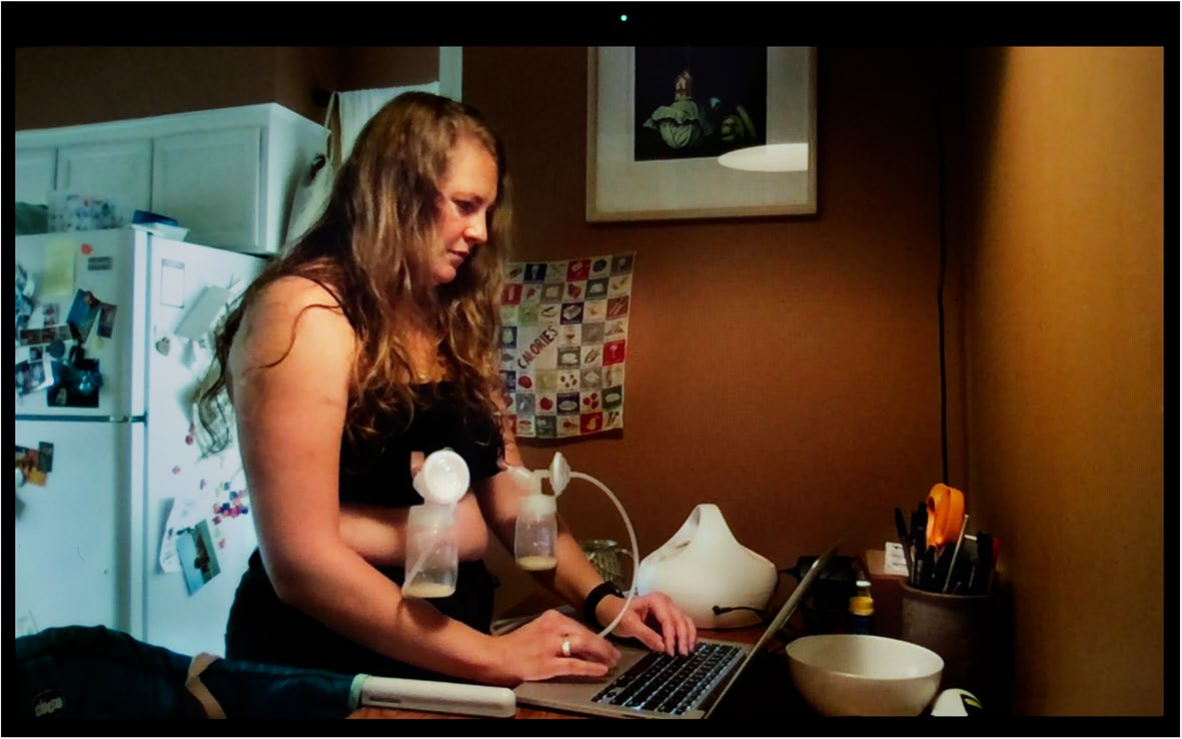
Greta

For White breastfeeding mother and teleworker Greta (Fig. 16), online public lactation proved more fraught than in-person public lactation. Before the pandemic,

nursing in public and nursing around other people was a priority—not that I was ever intending to shove it down people’s throats or be flamboyant about it, but I think that it’s cool and you have to feed your baby when your baby’s hungry, no matter where you are, and it’s not a big deal, and we should all recognize that.”

Now that she was home working remotely, she turned off the camera during meetings—and sometimes her mic too—when feeding at the breast or pumping. But to be as efficient as possible, she carried on with her work assignments as she expressed her milk, as seen in Botz’ picture. Similarly, Iris (Fig. 13) disabled her camera when she pumped during work meetings.

By contrast, Valentina (Fig. 15) kept her camera on when nursing during faculty and staff meetings. Bethany (Fig. 3) did too, though she “tilts it up” so that her feeding at the breast is barely noticeable (but still hearable). White dance artist and body worker Ailey, who transitioned to teaching movement classes online to an audience predominantly composed of adult women, stated, “if I’m in Zoom and I needed to breastfeed, I would breastfeed.... My baby’s, like, very mobile now, so he often just, like, stands up and kind of crawls over and pulls my shirt down, which is what he’s kind of doing right now.” If breastfeeding in plain sight while working remotely was not experienced as problematic for these mothers, Greta and Iris only felt comfortable expressing milk off-screen. This difference reflects American culture’s growing acceptance of feeding at the breast in public but the continued perception of milk expression as a practice that should be kept hidden, as will be discussed below.

### Human Milk—“Human connection”

The final theme we identified is that at a time when they felt lonely and isolated, parents saw human milk as a “human connection,” a link to others—a “liquid bridge” to use Tanya Cassidy’s expression [35]. With multiple crises raging—health, racial, political, economic, environmental—many parents yearned for the emotional and physical connection lactation can foster.

As White lactation consultant Katie shared,

I’ve gotten three texts in the last week from people I know who are isolating with their breastfeeding toddler, and they’re like, “All he wants to do is nurse all day long.” . . . Kids are so perceptive, and they know when we’re stressed out or when we’re having big emotions, and so they respond by wanting connection, by increasing their attachment. And…it’s normal, but it can be overwhelming, and it doesn’t mean that it’s easy.

Lactation in quarantine proved a strange mixture of loneliness and bond in which human milk feeding connects parents and children, but also families and friends through the peer sharing of human milk—the practice of donating and receiving human milk directly from family to family without resorting to an intermediary institution such as a human milk bank.

Some of the parents reported producing and giving away more milk than they anticipated. They were spending more time at home with their children and thus had less (or no) need for expressed milk to feed them in their absence. They wanted to help out and empty their freezers to make room for solid foods at a time when frequent grocery trips were discouraged. Greta recounts, “I had built up this supply thinking I would go back to the office, and it was taking over our freezer. I think I donated—the first donation was 200 oz or something.” For Brielle, the pandemic was a motivation to relactate so that she could donate milk and boost her own son’s immunity. She had been missing lactation since she had weaned him a few months earlier. In early March 2020, while at a breastfeeding conference, she received an email from a panicked new mother who wrote, “I’m supplementing, and they’re saying that there’s not going to be enough formula if we go into a lockdown. I don’t know what to do.” Brielle “came back home from the conference that day and said, ‘I’m going to relactate. We are going to do it.’” In just twenty days, she was able to build up her supply back to 20 oz per day, allowing her to donate to local parents as well as military moms.

**Fig. 17:**
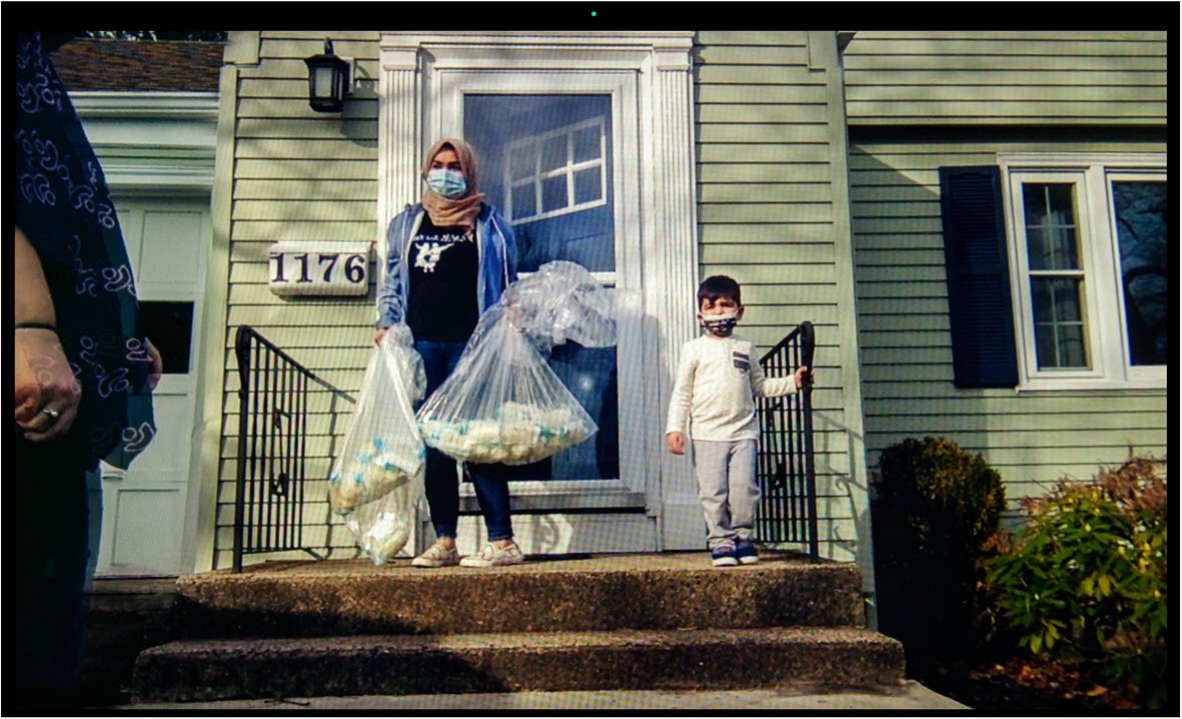
Marwa

Before the pandemic, informal human milk sharing used to be a venue for various forms of relationships among families, such as socializing and community organizing [35, 36]. With social distancing requirements in place, milk exchanges moved to porches and empty parking lots with no (or minimal) physical contact. Yet, they continued to be prized opportunities for connection. Stay-at-home mother of two Marwa (Fig. 17), who identifies as Arab and Muslim, has donated hundreds of ounces of milk to a devout Christian mother. They meet “masked up” on her front door or driveway. They do not get the chance to talk much, but Marwa appreciates that they share a “similar faith,” giving them “a bit of a connection.” In this picture, Marwa can be seen with her older son. She is holding three large bags full of her bottled expressed milk ready to hand off to the receiving mother, who is standing in the corner of the picture’s foreground.

When the pandemic hit, Aimee and her husband (Fig. 7), who live in a large urban center, purchased a car, which proved key to collect donor milk around the city and beyond when she had to supplement and then quit nursing. Initially, “it was like going out to get supplies during a zombie apocalypse.” But with some donors, they “were out in the yard together with masks on”—a rare occasion to meet in person and socialize. The picture shows her back home feeding her baby donor human milk while wearing her Black Lives Matter tee-shirt.

Socially distanced milk sharing can represent a unique way to maintain an emotional connection in some instances. Ittai, a White academic, and his husband had a daughter days before COVID-19 was declared a pandemic. Their gestational surrogate volunteered to donate her milk, but she lived on an island connected to their town via a ferry service that was curtailed due to government restrictions to curb the spread of the virus. Instead of visiting with one another regularly, as they had hoped for to maintain their close connection, they ended up sharing milk via an uncommon type of transportation. Initially, Ittai looked into using traditional shipping carriers, but “they were all difficult now or expensive or it wasn’t possible.” Desperate, he turned to a seaplane company that usually operates a touristic and high-end commuting route. When he called, he thought, “there is no way that they are going to do it... But they were very skilled about it and very laidback, easy. They said, ‘Of course, bring it,’ and gave some instructions. It wasn’t even as expensive as I thought it was going to be.” When he collected the first batch from the airport, it was the first time he had left the house after three weeks of strict quarantine. It felt surreal. Attired with gloves and a mask, he walked empty streets to the terminal, “there was no one in line, of course, because who would even use them [seaplanes] these days?”.

Milk sharing—be it peer-to-peer or through milk banks—epitomizes the pandemic’s twin effect of concurrently bringing people closer together by exchanging a bodily fluid and keeping them apart by effectuating this exchange at a distance. When community transmission rates of COVID-19 are high, sharing the same room, water bottle, or breath of air can put one at risk of infection with SARS-CoV-2, but human milk can to circulate among families with little risk so long as the exchange itself is socially distanced [37].

## Discussion

This article analyzed the lived experiences and representation of human milk feeding during the pandemic among a small sample of parents and lactation professionals. Our main finding is that while the pandemic had mixed effects on lactation, it exposed continuing challenges of infant feeding in a society that does too little to support young parents, generating new forms of (in)visibility for lactating labor. In highlighting these different effects of the pandemic, the article engages with several bodies of research on women’s work [34, 38], mothering [38, 39], and lactation [40–45].

On the one hand, the pandemic may have exacerbated preexisting inequities in lactation. It made it harder for under-resourced parents to initiate and maintain their lactation when they were separated from their children, cut off from their support system, sick, constrained to work stressful or unaccommodating shifts as essential workers, had diminished access to skilled and in-person medical care and lactation counseling, among other factors [7]. On the other hand, some of the very features of the crisis that hindered lactation in some contexts, in particular social isolation, proved beneficial in others. Some of the homebound parents may have benefitted from being able to feed at the breast on demand uninterrupted—or to pump in the comfort of their homes—even if their intensified lactation was an additional job on top of teleworking, homeschooling, homecare, and childcare, among other obligations [2]. Taken together these threads reveal that the pandemic exasperated the contradictions of motherhood and the contested nature of lactation in US society and culture.

### Idealized Motherhood and lactation

Lactation is viewed as a public health issue [46], putting parents under intense social pressure to breastfeed for at least six months. Yet, little concrete legal and policy support exists to support families in the United States, turning lactation into a burden for individuals to bear more or less alone [47]. It is expected to be done for free, without complaint, and in private. As a result, lactation has become a lightning rod in contemporary American society, in part because it embodies—often literally—the complexities and contradictions that many people feel about parenthood and specifically about the entanglement of the biological and the cultural.

Contrary to the saying “It takes a village” brought up over and over by participants in this study, dominant societal norms in the United States construct motherhood as a (classed, gendered, raced) individualist enterprise. In the 1990s, Sharon Hays identified the ideology of intensive motherhood, characterized by conflicting demands on working mothers and ideas about how they are to behave, and most prevalent among middle-class White women [39]. According to this ideology, the mother is a parenting expert completely and constantly devoted to her children. A couple of decades later, Dawn Marie Dow argued that middle and upper-middle-class African American mothers share some of these standards, but also view paid employment as a duty of motherhood, in addition to having to develop a repertoire of strategies to respond to the systemic racism shaping their lives [48]. American mothers are expected to be multitasking, self-reliant individuals who prioritize the health and well-being of their children, including by feeding them human milk, with hardly any government support.

Though the pandemic undoubtedly negatively impacted millions of Americans economically, data from the Bureau of Labor Statistics shows that women disproportionately felt the force of these losses [49]. In the month of April 2020 alone, women accounted for 55% of the 20.5 million jobs lost [50]. With the closure of daycares, schools, and other childcare, eldercare, and disability resources, women also took on the major part of unpaid family care. These disruptions lasted months and some of them are ongoing at the time of writing. For women of color, the picture is even starker, as they were the hardest hit by pandemic joblessness and business closures [51]. A higher proportion of Black and Latinx women are essential workers in the healthcare, childcare, grocery, warehouse, and delivery sectors, but many were also employed in hard-hit sectors such as hospitality and retail [52]. As Aimee (Fig. 7) summed it up,

White women in America have more socioeconomic power—you have more socioeconomic power; you’re more likely to succeed in breastfeeding . . . you have the time; you have the money for lactation consultants; you have the money for pump machines and supplements and all that stuff. . . . I did notice that women of color have slower breastfeeding outcomes.

In the first year of the pandemic, as existing interpersonal and institutional support systems broke down, mothers still held themselves to the model of intensive motherhood, dedicating themselves to their children physically, emotionally, and psychologically. All the parents in our sample fed their babies human milk while carrying the weight of multiple other paid and unpaid responsibilities. Bethany (Fig. 3) poignantly exemplifies parents’ internalized view of idealized motherhood when she said about her parenting and breastfeeding during the pandemic, “I wanted to do it right. I wanted to be perfect.” For some parents, being confined with their children was affirming. Quarantine liberated them from the guilt they feel as part of what Caitlyn Collins has identified as “a type of internalized oppression,... [a] regulatory mechanism by which intensive mothering discourses reproduce mothers’ feelings of inadequacy” [53]. They were now with their children 24/7, no longer susceptible to pangs of guilt or shame at leaving the house and/or dropping them off at daycare. Valentina (Fig. 15) jokingly quoted an online meme circulating at the height of the pandemic, “my dog is so overjoyed I’m here all the time,” before adding, “I think she [her toddler] has the same sentiment.” Meanwhile, for other parents (or for the same parents at different times), the new parenting (and other) demands resulting from crisis meant that they had less time and energy to dedicate to their children. They had to loosen their parenting, cleaning, working, and other standards, fueling feelings of inadequacy and failure.

As a form of gendered, embodied labor at the cusp of care work and dirty work, lactation has oscillated in status depending on the context and period. In the contemporary United States, lactation is not widely recognized as work deserving of recognition and compensation [13]. Economist Nancy Folbre [54] has shown that American society does not value or reward care work like other types of labor. Care work is conflated with women and motherhood and devalued as such [55]. Lactation is a paradigmatic example of such care work, historically provided by impoverished immigrant and/or single women, servants, enslaved women, or women in their unpaid roles as wives and mothers [56]. The government, employers, medical providers, and insurers free ride on parents’ lactation [57]. The economic worth of human milk is barely acknowledged, like much of women’s reproductive and affective labor. Standard accounting practices include in Gross Domestic Product (GDP) calculations infant formula and cow’s milk produced and consumed on farms, but not human milk [58]. Parents who breastfeed do it at great personal cost—those “who breastfeed for six months or longer suffer more severe and more prolonged earnings losses than do mothers who breastfeed for shorter durations or not at all” [57, 59]. The longer people breastfeed, the more likely they are to be non-employed or to work fewer hours. Conversely, paid maternity leave increases the odds of lactation initiation and longer breastfeeding [58].

**Fig. 18:**
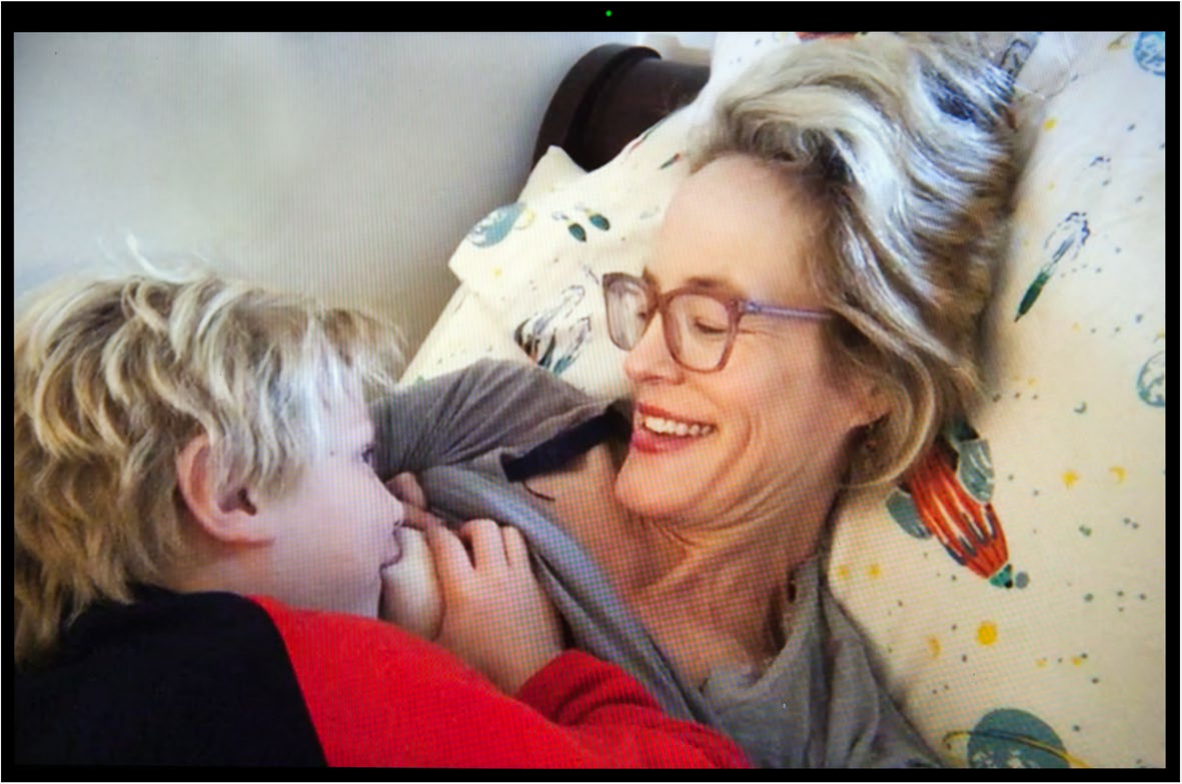
Claire

It remains to be seen whether parents who breastfed their children throughout the pandemic were more likely—or not—to lose on economic and professional opportunities in the long and short term. To be sure, giving birth and lactating during the pandemic came with increased psychological and physical risks. The anxiety of delivering during a health crisis combined with isolation and the retreat of social services contributed to a surge in “Perinatal Mood and Anxiety Disorders” (which include postpartum depression) according to White breast surgeon and lactation consultant Claire (Fig. 18). An essential worker and breastfeeding mother herself, she recalls a week in the early spring when she came home on a Friday realizing that she had encountered a “serious mental health problem every single day with patients—to the point where I was like, ‘Wow. I need a break.’” These disorders can translate into over-pumping milk or obsessively storing milk. Anahita, who grew up experiencing “periods of starvation as a kid” confided that she “was pumping like crazy in the early days to both get my production up and to have a stockpile of frozen breastmilk because—I didn’t connect this until later—I thought: my baby needs resources. What if the resources go away?” News reports about infant formula shortages made lactation initiation and continuation feel even more critical and potentially stressful to new parents [9].

In some cases, excessive pumping can lead to serious harms such as abscesses and hyperlactation, which are hard to diagnose and treat with reduced face-to-face support, as Bethany (Fig. 3) and Theadora (Fig. 10) experienced firsthand. Bethany’s latch difficulties could not be adequately assessed remotely, leading to nipple injury, and a life-threatening infection, as she had breast implants, which had to be removed. As for Theadora, an immigrant with no employer-provided health insurance, she could only see providers who accepted an emergency medical care insurance program for non-citizens, constantly worrying about her eligibility running out. When her left breast became painful, she could not secure in-person visits with lactation and medical professionals to figure out whether she had a plugged duct or a more serious condition such as mastitis. Misdiagnosis led to a severe infection, an abscess, a fistula, numbing pain for weeks, five rounds of antibiotics, several procedures to drain the accumulated fluid in her breast, having to use donor human milk to feed her baby, depression, and a series of miserable encounters with medical professionals who urged her to quit breastfeeding or else, they incorrectly claimed, she would need to get a mastectomy. Bethany too was inaccurately told by a surgeon that she had to “stop breastfeeding” otherwise her implants could not be surgically removed. She was “devastated,” and remembers telling her husband that rather than getting the surgery, she would “sacrifice my body” to continue breastfeeding until their baby turned one. She eventually found a competent and compassionate surgeon who performed the surgery without interfering with her breastfeeding.

In short, the pandemic intensified the individualistic construction of parenting and infant feeding as an undertaking run and managed by one primary person—the mother. It also contributed to further invisibilizing lactating labor—except when it came to shaming it.

### The (in)visibility of lactation

Paradoxically, the pandemic made lactation simultaneously more and less visible. In the age of social distancing and stay-at-home orders, public nursing waned from our collective sight. The image of a woman feeding her baby at the breast is a paradigmatic symbol of communion and closeness across cultures from Isis to Parvati to Mary, all frequently depicted nursing their divine children. This iconic vision is seldom to be seen when families are quarantined. For the same reason, the still marginal, but growing trend of expressing milk in public has halted. With the crisis, lactation returned to the private, domestic space, where it has long been thought to belong [60–62].

At the same time, a novel form of “public” lactation is developing on videoconferencing platforms, which function in many ways as the new public space in which people socialize, work, engage in politics, protest, exercise, experience art, and more. Some parents need or choose to nurse at the breast or to express milk visibly while online. This emerging practice is not always met with approval, as illustrated by the shaming on Reddit of a woman who kept her mic and camera on while breastfeeding during an online work meeting [63].

Telework in particular exposes the contested nature of lactation as a quasi-maternal duty which is expected to be accomplished behind closed doors. What other activity is considered normatively desirable but not acceptable to be performed in full view? Glorified as “liquid gold” in medical and scientific discourse, in practice, like other (especially female-coded) discharges, human milk is often treated with a combination of fear and disgust [64]. Lactation is coded as a “private” activity to confine to the home and other secluded spaces such as the increasingly popular lactation rooms and pods. The pandemic reinforced this coding by displacing most of the lactation we have grown accustomed to witnessing in public spaces to domestic spaces. Nicole Owens and colleagues have shown that any space can be defined as public depending on the presence of certain others [65]. The pandemic provides a specific instance of this relational definition of space, challenging the traditional public–private divide, by transforming the home into a semi-public space through video-conferencing and teleworking. The home, which was already a “fragile site[] for breastfeeding since one person’s presence can transform the entire place’s public/private designation” [65], is even more fraught now that it has been opened up to the world via digital technologies.

Is a culture of online public lactation developing and, if so, is it favorable to parents and their children? Even with the 2010 Affordable Care Act’s mandate that employers offer lactation breaks, in the past decade, workers across the country have continued to experience discrimination, seeing their requests for breastfeeding and milk expression denied or rewarded by dismissal, or lacking time and adequate spaces. Breastfeeding in public has slowly gained acceptance—since 2018 all the 50 states have legalized it [66]. But by and large *expressing* milk in public continues to be seen as a disgusting bodily function analogous to public urination or sex. Only a handful states explicitly exempt it from their indecent exposure and obscenity laws [66]. Moreover, so far laws and policies have not addressed the issue of workers lactating while telecommuting. Can they only feed at the breast or express milk during specific, preestablished lactation breaks? Or are they entitled to lactate at any time and for however long they want given that they are working from home? Must they disconnect from their online assignments or turn off their mics and cameras or can they conspicuously engage in lactation labor? This is a grey area in which different practices and preferences are emerging.

With the pandemic, virtual rooms have become a space of choice for shared experiences, yet participants typically access them from physical spaces considered private. Professional video conferences in particular call for workers to relocate their cognitive and emotional presence into metaphoric rooms with other employees. But how can people turn into disembodied workers when submerged by care work that cannot always be segregated from their physical space?

If the 2000s were the decade of the “brelfie,” that is, of carefully curated breastfeeding selfies shared online [67], the early 2020s could be dubbed the years of “LacZoom” to connote the different ways and degrees to which people expose their lactation on videoconferencing software. Unlike brelfies, which risk censorship on social media such as Facebook or Instagram for violating indecency and nudity policies [68], LacZoom happens in real time, reducing the chance that it will be blocked. Nevertheless, much like breastfeeding in public out in the world is expected to be low profile—a few states actually require discretion [69, 70]—online lactation is tolerated when it is not too visible.

It remains to be seen whether the emergence of video conferencing apps as our new pandemic public space will contribute to normalize lactation in public or further consign it to the private sphere. There are similarities here with the increased availability of lactation rooms and pods in the workplace as well as in public or semi-public spaces. This development has been applauded as providing a modicum of comfort and privacy—a welcome alternative to pumping in bathroom stalls. But what is the message sent by these facilities or the possibility of turning off cameras and mics? Is it, “We support you and your children in your lactation journey” or, “We expect you to produce this highly valuable liquid concealed?” The paradox of the unfolding crisis is that parents hear both communications at the same time, redoubling their struggle. The good mother is the lactating mother, who earns symbolic capital from her milk production, yet she is shamed for lactating too visibly or for requesting accommodations to lactate privately.

With the rise of online spaces as public spaces, lactation has acquired a newfound visibility. But will it change the social meaning, status, and location of the activity in the long haul? Somehow breastfeeding or expressing milk online may be less powerful than in the offline “real” world, as illustrated by Valentina’s (Fig. 15) experience. Many of her colleagues did not seem to notice when she was breastfeeding her toddler on Zoom calls. For some, especially other mothers, there seemed to be no doubt as to what was occurring, while others may have had no clue, or be left to wonder, “Could she be nursing?” It is unlikely that Valentina would have gone unnoticed if she had breastfed at an in-person meeting, highlighting the distinctiveness of remote interactions. In some ways, videoconferencing platforms allow more control over how others see us—after all we constantly monitor ourselves in the self-view functions. The ability to see the image of one’s own lactation may feel empowering and healing. Embodied maternal subjects can finally fully cohere with their social and professional beings. In other ways, our private spaces and lives are both more exposed than before and less well-defined in a way that could hamper a broader social recognition of the labor involved in lactation.

## Conclusion

The pandemic restrictions have cut deeply into our existence, providing an occasion to think carefully about how parents and their children are treated. At a time when self-sufficiency is seen as a key element of disaster preparedness and pandemic response, communities around the nation, and the globe, have looked into starting gardens, making soap, sewing clothes, and preserving food. Some parents have resorted to their own bodies as a source of food sovereignty by increasing their milk production, relactating, and/or donating their milk. But not everyone is able to engage in such practices. Through interviews and photographs, this article endeavored to expose the lived experiences of human milk feeding during the pandemic as well as some of the fundamental inequities reinforced by the crisis. Many observers over the last couple of years have noted how the pandemic reflects, exposes, or refracts existing social structures and inequalities. It functions as a magnifying glass highlighting the inequities of our current cultural, economic, and legal order. In the United States, most people feed their children human milk against all odds in the absence of universal basic income, paid parental leave for at least 6 months, paid lactation leaves and breaks, affordable housing, universal health care, equal access to high-quality, non-discriminatory, and culturally appropriate healthcare (including lactation support), sliding fee childcare programs, and more.

One lesson of the pandemic for policy and lawmakers may be that to adequately support lactation, they should take cues from the families who had positive experiences during the crisis. These are typically the well-resourced households in which the lactating parent is either not working outside the home or telecommuting while receiving support from their partner and others. These parents had the opportunity to engage in the types of practices known to support lactation such as increased time at home, fewer interruptions, and access to lactation support. In some ways, they observed traditional postpartum customs and rituals known in some cultures as confinement [71]. These practices are characterized by a period of rest, care for the birthing parent and their infant, social isolation, and prescribed foods to be eaten. Of course, “confinement” is a term also used to denote quarantine. For all new parents to take these lessons from confinement to regular life—whatever that looks like and whenever it comes—we need core legal and policy supports.

## Data Availability

The interview data is not available to protect the confidentiality of the participants and the photographs are the intellectual property of artist Corinne Botz.
